# Interventions to change vaping harm perceptions and associations between harm perceptions and vaping and smoking behaviours: A systematic review

**DOI:** 10.1111/add.70129

**Published:** 2025-07-25

**Authors:** Katherine East, Erikas Simonavičius, Eve V. Taylor, Leonie Brose, Deborah Robson, Ann McNeill

**Affiliations:** ^1^ Department of Primary Care and Public Health, Brighton and Sussex Medical School University of Brighton and University of Sussex Brighton UK; ^2^ National Addiction Centre, Institute of Psychiatry, Psychology and Neuroscience King's College London London UK; ^3^ Department of Behavioural Science and Health University College London London UK

**Keywords:** adults, perceptions, smoking, systematic review, vaping, young people

## Abstract

**Aims:**

To synthesize and describe the evidence from among young people and adults to assess: (i) what interventions have been effective in changing vaping harm perceptions; and (ii) to what extent vaping harm perceptions predict any changes in vaping and smoking behaviours.

**Methods:**

Systematic review searching five databases (Embase, PsycINFO, Medline, CINAHL, Scopus) from January 2007 to January 2023. Eligible studies reported quantitative data with >1 time point among young people (sample majority aged <18 years) or adults (sample majority aged ≥18 years). Interventions were considered if they communicated vaping harms, categorized as relative (vaping vs smoking) or absolute (vaping vs not vaping). Outcomes were changes in: (i) vaping (absolute, relative) or nicotine harm perceptions; (ii) vaping or smoking behaviours. Evidence was synthesized narratively.

**Results:**

Eighty‐five articles were included, of which 46 assessed interventions to change vaping harm perceptions and 39 assessed associations between harm perceptions and subsequent vaping/smoking behaviours. All studies among young people and most among adults were from the USA. Interventions aimed at young people typically communicated that vaping and nicotine are harmful/addictive (absolute harms), often in the form of educational programmes and media campaigns. Interventions aimed at adults typically communicated that vaping is harmful but less harmful than smoking (relative harms), often via written materials and educational workshops. In addition to methodological and analytical heterogeneity, risk of bias was high; hence findings should be interpreted with caution. Generally, interventions appeared to be effective in changing perceptions that reflected the intervention content among young people (12/14 studies) and adults (24/32 studies), at least in the short‐term (38/46 studies only assessed the outcome pre‐ and immediately post‐intervention). Interventions communicating that vaping is harmful and addictive (absolute harms) increased perceptions that vaping is harmful and addictive among young people (12/14) and adults (16/23 studies) and also increased the misperception that vaping is as harmful as smoking (relative harm) among young people (2/2 studies) and adults (5/8 studies). There was also some evidence that both absolute and relative harm perceptions predicted vaping and smoking behaviours, such that perceiving vaping as harmful deterred vaping among both young people (8/9 studies) and adults (4/7 studies), while misperceiving vaping as equally/more harmful than smoking prevented adults from quitting smoking (5/6 studies).

**Conclusions:**

Interventions to change vaping harm perceptions appear to be effective. Vaping harm perceptions appear to predict vaping and smoking behaviours.

## INTRODUCTION

Vaping carries some risks but is less harmful than smoking [1, 2, 3]. However, misperceptions of the health harms of vaping are increasing in many countries [4, 5, 6, 7, 8, 9]. In England in 2024, 85% of adults who smoked inaccurately perceived that vaping is equally or more harmful than smoking or did not know, up from 59% in 2014 [9]. In the USA in 2019, 16% of adults accurately perceived that vaping is less harmful than smoking, down from 42% in 2013 [8]. Nicotine misperceptions are also common [4, 5, 10]; in 2021, only 11% of adults who smoked in England knew that nicotine contributes none or a small amount of the health harms from smoking [5]. Similar patterns are also seen among young people [5, 7, 8]. Little is known about how these misperceptions can be corrected, but doing so is critical because they could deter adults who smoke from switching to a less harmful nicotine product [1, 5]. However, vaping is not risk free and so should be discouraged among young people and non‐smokers [1, 5].

Systematic reviews have found that exposure to risk messages about vaping are associated with vaping harm perceptions and intentions to vape [11, 12], and that, among young people, vaping harm perceptions are associated with ever having vaped [13]. Specifically, one review from 2020 concluded that messages communicating that vaping is less harmful than smoking improved the accuracy of vaping harm perceptions and increased intentions to quit smoking and/or switch to vaping [11]. The review identified 31 experimental and cross‐sectional studies, all from the USA or UK, and none examined changes in vaping or smoking behaviours [11]. A 2024 review of 39 studies among young people (with the majority conducted in the USA) identified numerous interventions highlighting vaping risks, and these increased perceptions that vaping is harmful and decreased intentions to vape [12]. However, most studies were limited by cross‐sectional designs or short follow‐up periods [12] and none considered adults. A 2022 meta‐analysis of 20 studies from across multiple countries (half from the USA, one‐fifth from Asia) found that young people who have ever (vs never) vaped were more likely to perceive vaping as not harmful, including less harmful than smoking [13]. However, most studies were cross‐sectional and the review did not consider more frequent vaping, changes in smoking or adults [13].

To our knowledge, there have been no evidence reviews of the longitudinal impact of vaping harm perceptions on subsequent vaping and smoking among both young people and adults. Studying both age groups is important because interventions should balance encouraging adults who smoke to switch to vaping with deterring vaping in young people.

This systematic review therefore aims to assess, among young people and adults: (i) what interventions have been effective in changing vaping harm perceptions; and (ii) to what extent are vaping harm perceptions predictive of any changes in vaping and smoking behaviours.

## METHODS

### Protocol

The protocol was registered (PROSPERO ID: 247890) [14] and the Preferred Reporting Items for Systematic Reviews and Meta‐Analyses (PRISMA) guidelines were followed (Table S1). This review extends prior vaping evidence reviews in 2022 and 2024 [1, 5].

### Search

Embase, PsycINFO, Medline, CINAHL and Scopus were searched from 1 January 2007 (the year e‐cigarettes were introduced to the UK and US markets) to 24 January 2023. In PROSPERO, the end date was set to 1 August 2021, but has since been updated to 24 January 23. See the supporting information for the search strategy, which was informed by prior work [15, 16, 17, 18, 19].

### Inclusion criteria

*Population*. Any people, any age.

*Language*. English, French or German.

*Literature*. Peer‐reviewed publications. Excluded grey literature and conference abstracts.

*Design*. Quantitative data with more than one time point. For research question 1 (RQ1), longitudinal (i.e. the same participants assessed at more than one time point), repeated cross‐sectional, experimental/quasi‐experimental, trials, surveys and observational studies that examined interventions involving communication/messaging of the harms of vaping and changes in vaping harm perceptions (within‐ or between‐person) were included. For RQ2, studies that examined longitudinal associations between vaping harm perceptions and any subsequent changes in vaping and smoking behaviours within‐person were included.

*RQ1 exposure*. Interventions involving communication/messaging of vaping harms or risks.

*RQ1 outcome*. Changes in vaping harm perceptions, categorized as:

1. Relative – harms of vaping compared with smoking, including health effects or addiction (e.g. vaping is less harmful than smoking, is less addictive than smoking). Based on current evidence [5], we classified the perception that vaping is less harmful than smoking as ‘accurate’ and equally/more harmful than smoking as ‘inaccurate’.
2. Absolute – harms of vaping compared with not vaping, including health effects or addiction (e.g. vaping is harmful, causes diseases, is addictive).
3. Nicotine – harms of nicotine use compared with no nicotine use, including health effects or addiction (e.g. nicotine is harmful, is addictive).

Changes could be measured within‐person (e.g. experiments, longitudinal surveys) or at the population level (e.g. repeated cross‐sectional surveys), and must have been measured before/concurrently and after the intervention, with any changes reported. We excluded harm perceptions of vaping cannabis/illicit drugs and harm perceptions as a reason for vaping (i.e. not harm perceptions per se, but vaping because of perceived harms).

*RQ2 exposure*. Vaping harm perceptions (defined above).

*RQ2 outcome*. Longitudinal changes in vaping/smoking behaviours, assessed within‐person (e.g. initiation/uptake, cessation/reduction, switching from smoking to vaping). Behaviours must have been measured before/concurrently and after harm perceptions, and changes reported.

### Screening

**FIGURE 1:**
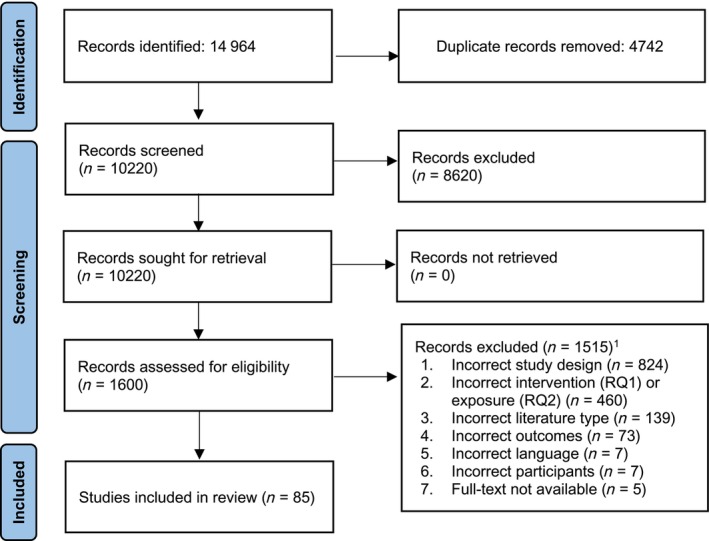
Preferred reporting items for systematic reviews and meta‐analyses (PRISMA) flow diagram describing article selection.

Two authors independently screened all titles/abstracts and full texts in Covidence (Covidence, Melbourne, Australia) [20]. Agreement was fair (title/abstract agreement = 87.6%; Cohen's kappa = 0.38; full‐text agreement = 86.5%; Cohen's kappa = 0.35). Most disagreements were because of poor intervention or measure descriptions or a lack of clarity over whether data were longitudinal. Title/abstract disagreements were automatically screened at full‐text level. Full‐text disagreements were resolved through discussion, consulting a third reviewer and, where necessary, contacting authors for additional information. Reason for exclusion disagreements were resolved according to the hierarchy of exclusion criteria (Figure 1), taking the lowest number selected. Where information for determining eligibility was missing, the authors of the article were contacted. Articles where authors did not provide the necessary information were excluded.

### Data extraction

Data were extracted in Covidence [20] by one author and 10 (8.5%) articles were checked by a second author. The quality was considered good and so further checks were not performed. For the data extraction form, see the Supporting information.

### Risk of bias

Risk of bias was assessed by one author using the Cochrane Risk of Bias (RoB) tool (randomized studies) [21], Risk of Bias in Non‐randomized Studies of Interventions tool (ROBINS‐I) [22], adapted five‐star Newcastle–Ottawa Scale (NOS) (cohort studies) [23, 24] and adapted eight‐star NOS (repeat cross‐sectional studies) [25].

### Analyses

Findings were narratively synthesized because of methodological and analytical heterogeneity, as expected [14]. We grouped articles by age group [young people (sample majority aged <18 years), adults (sample majority aged ≥18 years), young adults (sample majority aged 18–34 years, mean age <26 years or undergraduate students)]. We also grouped harm perceptions and interventions into three categories (relative, absolute, nicotine) and further categorized the ‘relative’ category as ‘accurate’ or ‘inaccurate’ (defined above). Where articles included findings from the same study, all articles were included and any overlap was described narratively.

## RESULTS

The search identified 14 964 records, of which 85 were included (Figure 1). Of these, 46 addressed RQ1 (interventions to change vaping harm perceptions) [26, 27, 28, 29, 30, 31, 32, 33, 34, 35, 36, 37, 38, 39, 40, 41, 42, 43, 44, 45, 46, 47, 48, 49, 50, 51, 52, 53, 54, 55, 56, 57, 58, 59, 60, 61, 62, 63, 64, 65, 66, 67, 68, 69, 70, 71] and 39 assessed RQ2 (whether harm perceptions predict subsequent vaping and/or smoking behaviours) [72, 73, 74, 75, 76, 77, 78, 79, 80, 81, 82, 83, 84, 85, 86, 87, 88, 89, 90, 91, 92, 93, 94, 95, 96, 97, 98, 99, 100, 101, 102, 103, 104, 105, 106, 107, 108, 109, 110]. Table 1 presents a summary of the results.

**TABLE 1:** Summary of results.

	Youths	Adults
**Research question 1**	**14 articles in total**	**32 articles in total**
Interventions and absolute harm perceptions	12 of 14 articles found evidence of associations. Specifically, the perception that vaping is harmful was increased by interventions communicating that vaping is harmful, addictive and/or equally/more harmful than smoking	16 of 23 articles found evidence of associations. Specifically, the perception that vaping is harmful was increased by interventions communicating that vaping is harmful (sometimes also including relative and/or nicotine harms)
Interventions and relative harm perceptions	2 of 2 articles found evidence of associations. Specifically, the inaccurate perception that vaping is equally/more harmful than smoking was increased by interventions communicating that vaping and nicotine are harmful	10 of 16 articles found evidence of associations. Specifically, the accurate perception that vaping is less harmful than smoking was increased by interventions communicating that vaping is less harmful than smoking and can help people quit smoking (sometimes also including information about nicotine). The misperception that vaping is equally/more harmful than smoking was increased by interventions communicating that vaping is harmful or as/more harmful than smoking
Interventions and nicotine harm perceptions	3 of 4 articles found evidence of associations. Specifically, perceptions that nicotine is harmful and addictive were increased by interventions communicating that vaping is harmful and nicotine is addictive	6 of 7 articles found evidence of associations. Specifically, perceptions that nicotine is harmful and addictive were increased by interventions communicating that vaping and/or nicotine are harmful and addictive
**Research question 2**	**14 articles in total**	**25 articles in total**
Absolute harm perceptions and vaping	8 of 9 articles found evidence of associations. Specifically, perceiving lower harms from vaping predicted the initiation and escalation of vaping	4 of 7 articles found evidence of associations. Specifically, perceiving lower harms from vaping predicted the initiation and escalation of vaping, while perceiving vaping as harmful predicted quitting vaping
Absolute harm perceptions and smoking	1 of 3 articles found evidence of associations. Specifically, perceiving more harm from vaping/smoking (combined) predicted not smoking in the past 30 days	0 of 2 articles found evidence of associations
Relative harm perceptions and vaping	4 of 7 articles found evidence of associations. Specifically, perceiving vaping as less harmful than smoking predicted the initiation and escalation of vaping	13 of 16 articles found evidence of associations. Specifically, perceiving vaping as less harmful than smoking predicted the initiation and escalation of vaping, switching from smoking to vaping (i.e. quitting smoking), using vapes to quit smoking and past 30‐day vaping
Relative harm perceptions and smoking	0 of 2 articles found evidence of associations	5 of 6 articles found evidence of associations. Specifically, perceiving vaping as less harmful than smoking predicted switching from smoking to vaping (i.e. quitting smoking) and reductions in smoking. Perceiving vaping as equally/more harmful as smoking predicted a relapse to smoking
Nicotine harm perceptions and vaping	1 of 1 article found that perceiving nicotine in vapes as harmful predicted quitting vaping	1 of 3 articles found evidence of associations. This article found that lower perceptions that nicotine is harmful predicted past 30‐day vaping and vaping for quitting smoking
Nicotine harm perceptions and smoking	No studies were identified.	1 of 1 article found that perceiving nicotine as harmful predicted more quit attempts but less quit success

### RQ1: interventions to change vaping harm perceptions

#### Young people

Among young people, 14 articles assessed associations between interventions and changes in vaping harm perceptions (Table 2) [26, 27, 28, 29, 30, 31, 32, 33, 34, 35, 36, 37, 38, 39].

**TABLE 2:** Interventions communicating the harms of vaping and associations with changes in vaping harm perceptions (research question 1) among young people (*n* = 14 articles). Findings are sorted by number of outcomes assessed followed by finding an effect of the intervention on an outcome.

Ref., country	Analytic sample, design	Interventions (message)		Outcomes (harm perceptions): measurement and association with intervention			Risk of bias^b^
		Category^a^	Details and examples	Relative (accurate)^a^	Absolute^a^	Nicotine^a^	
Noar (2019) [26] USA	*n* = 61 Age 14–18 years One‐group experiment	Absolute Nicotine	Written information (e.g. ‘contains harmful chemicals. Poisonous if swallowed’, ‘e‐cigarettes and vaping devices contain nicotine. Nicotine is an addictive chemical’, ‘nicotine in e‐cigarettes and vaping devices may harm teen brain development’)	**✓** Perception that vaping is equally/more harmful than smoking increased	**✓** Perceptions of vaping harms (dangers, worry, health consequences, addiction, harmful chemicals, teen brain development increased	✘ No significant change in perception that e‐cigarettes contain addictive nicotine	ROBINS‐I: serious risk
Weser (2021) [27] USA	*n* = 285 Age 11–14 years Non‐randomized experiment	Absolute Nicotine	Virtual reality video game in which players work on skills to avoid vaping and smoking and learn vaping harms and nicotine risks (e.g. ‘One JUUL pod has the same amount of nicotine as a pack of cigarettes!’ and ‘JUUL has a very high level of nicotine and you can get addicted, FAST!’). The control was treatment‐as‐usual, but this was not defined	N/A	✓ Perceptions of vaping harms (e.g. people harm themselves when they vape) and addictiveness (e.g. vaping is difficult to quit) increased immediately after the intervention, and were sustained 6 months later, to a greater extent than the control	✓ Perceptions of nicotine harms (e.g. addiction to nicotine can rewire your brain) increased immediately after the intervention, and were sustained 6 months later, to a greater extent than the control	ROBINS‐I: serious risk
Gaiha (2022) [28] USA	*n* = 1031 School students (middle/high), mean age 14.4 years Non‐randomized experiment	Absolute Nicotine	Educational programme that included a 25‐minute presentation on vaping, either in‐person or virtually. Content was based on the Stanford Tobacco Prevention Toolkit and focused on vaping health effects, risks of second‐hand/third‐hand smoke, nicotine as being addictive and marketing strategies	N/A	✓ Perceptions of vaping harms (e.g. vaping can increase COVID‐19 risks, long‐term vaping effects are unknown, vaping increases risk of lung/heart disease, second‐hand aerosol contains cancerous chemicals) increased slightly, immediately after both the in‐person and virtual intervention conditions (but statistical contrasts were not reported)	✓ Perceptions of nicotine harms including addiction (e.g. nicotine makes most e‐cigarettes extremely addictive, the amount of nicotine in JUUL is equivalent to 1–2 cigarette packs, nicotine not harmless, changes brain chemistry) increased slightly, immediately after both the in‐person and virtual intervention conditions (but statistical contrasts were not reported)	ROBINS‐I: serious risk
MacMon‐egle (2022) [29] USA	*n*= 3354 Age 11–16 years Cohort study, 13‐month follow‐up	Absolute Nicotine	Mass media. Self‐reported exposure to ‘The Real Cost’ campaigns in the past 3 months (0–4; never–very often). The campaign focused on health consequences, including introducing chemicals into the blood stream and lung damage (‘epidemic’) and addiction (‘rehacked’)	N/A	**✓/**✘ Agreement increased for 3/9 ‘epidemic’ beliefs (vaping containing formaldehyde/is an epidemic/causes irreversible lung damage) and 1/6 non‐campaign‐specific beliefs (vaping will decrease my sports performance) with greater exposure	**✓/**✘ Agreement increased for 3/8 ‘rehacked’ beliefs (nicotine hacks/reprograms/changes your brain) after exposure	Low risk (3)
Merrill (2022) [30] USA	*n* = 10 758 School students aged 11–18 years One‐group experiment	Absolute	Educational Clearing The Vapor (CTV) programme – an interactive education programme involving videos, readings, quizzes, reflection activities about the harmful effects of vaping and understanding increases in teen vaping	✓ Perception that vaping is a safer alternative to smoking decreased after programme	✓ Perception that vaping has high risk of harm increased after programme	N/A	ROBINS‐I: serious risk
Gaiha (2021) [31] USA	*n* = 2889 School students One‐group experiment	Absolute Nicotine	Brief educational workshop on vaping, which included information about e‐cigarette types, contents, health effects, nicotine addiction, the tobacco industry's manipulation of young people through advertising and marketing and flavours	N/A	**✓** Perceptions of vaping harms and addictiveness (e‐cigarette smoke is not harmless, effects are unknown, e‐cigarettes are addictive) increased	N/A	ROBINS‐I: serious risk
Hieftje (2021) [32] USA	*n* = 560 Age 10–13 years One‐group experiment	Relative – inaccurate Absolute Nicotine	Video game, where players work on skills to avoid vaping/smoking and learn that vaping is equally/more harmful than smoking, harms health and nicotine is harmful (e.g. ‘e‐cigarettes produce second‐hand smoke, just like normal cigarettes’, ‘can damage a teenager's brain’)	N/A	**✓** Perceptions of vaping harms and addictiveness (e‐cigarettes are dangerous, harmful to health, difficult to quit, addictive) increased	N/A	ROBINS‐I: serious risk
Pentz (2019) [33] USA	*n* = 14 Age 11–14 years One‐group experiment	Relative – inaccurate Absolute Nicotine	Video game, as described above [32]	N/A	✓ Perceptions of vaping harms (people harm themselves when they use e‐cigarettes) increased	N/A	ROBINS‐I: serious risk
England (2021) [34] USA	*n* = 268 Age 11–19 years Randomized experiment	Relative – inaccurate Absolute Nicotine	Mass media campaign (‘Rethink Vape’) highlighting vaping risks including vaping is not less harmful than smoking, risk of specific diseases and nicotine addiction. Control was ‘Rev Your Bev’, a campaign about sugar in drinks	N/A	✓ Perceptions of vaping harms (e.g. seriously harms health, flavours linked to lung disease) increased after intervention but not in the control	N/A	RoB2: some concerns
Baker (2022) [35] USA	*n* = 72 School students (middle, typically aged 11–13 years) One‐group experiment	Absolute Nicotine	Educational programme, including lessons on vaping and nicotine risks and an interactive peer‐led group activity. Information was provided on vaping ingredients, health risks, policies and prevalence among teens, while the group activity focused on developing refusal skills (www.catchmybreath.org)	N/A	✓ Knowledge about vaping (11 questions, one about health risks) increased immediately after the intervention and this was sustained 3 months later	N/A	ROBINS‐I: serious risk
Noar (2022) [36] USA	*n* = 1514 Age 13–17 years (mean 15.2 years) Randomized experiment	Absolute Nicotine	Mass media. Exposure to ‘The Real Cost’ campaigns – 30‐second videos from the ‘epidemic’ (health consequences, including introducing chemicals into the blood stream and lung damage) or ‘rehacked’ campaigns (addiction) + control (3 × 30s neutral videos about vaping)	N/A	**✓** ‘The Real Cost’ increased perceptions of health risks (likely that vaping will damage your: body, lungs, brain), addiction risks (likely that if you vape you will be: addicted, controlled, unable to stop?) and negative attitudes (vaping is: bad, harmful, unenjoyable) relative to control; findings sustained over 3 weeks	N/A	RoB2: low risk
Weser (2021) [37] USA	*n* = 47 Mean age 14 years One‐group experiment	Absolute Nicotine	Virtual reality video game, as described above [27]. Examples of messages in this study are ‘toxic chemicals in e‐cigarettes that are known to cause cancer and other diseases’ and ‘Nicotine harms teen brain development and can even change the way the brain works’	N/A	**✓/×** Perceptions of vaping harms, incl. second‐hand exposure, were increased, but no change in perceived addiction or short‐term safety	N/A	ROBINS‐I: serious risk
Asdigian (2022) [38] USA	*n* = 130 School students (middle/high, typically aged 11–18 years) Non‐randomized experiment	Absolute Nicotine	Educational programme, including lessons on vaping and nicotine risks (although these are not described), the history of the tobacco and vaping industry, reasons why young people vape, resistance skills, and a task in which students develop and deliver a short narrative prevention video to peers, highlighting the risks of vaping (risks were not defined). The control was not taking part in the programme	N/A	✘ The programme group did not show a significantly larger change in the perception that vaping is harmful, relative to the control	N/A	ROBINS‐I: serious risk
Levy (2022) [39] USA	*n* = 4587 Age 12–17 years (median 13 years) Repeat cross‐sectional	Insufficient information	Educational programme. Screening and brief intervention (SBI) programmes in schools to prevent young people's substance use. Little information about what the intervention entailed or what was communicated. Control group were studies in school grades that did not implement SBI	N/A	✘ Intervention schools did not show a significantly larger change in the perception that vaping is risky to physical or mental health, relative to control schools, 3 months later	N/A	NOS: 3/8

✓ = evidence of associations (i.e. *P* < 0.05); ✓/✘ = mixed evidence of associations (e.g. for one subgroup only, or in unadjusted but not adjusted analyses); ✘ = lacking clear evidence for an effect (i.e. *P* > 0.05).

^a^ Categories of interventions and outcomes. Relative: harms of vaping compared with smoking, subcategorized as accurate (vaping is less harmful than smoking) and inaccurate (equally/more harmful than smoking). Absolute: harms of vaping compared with not vaping. Nicotine: harms of nicotine use compared with no nicotine use.

^b^ Risk of bias was assessed using different tools depending on the study design. The Cochrane Risk of Bias (RoB) tool for randomized studies, the Risk of Bias in Non‐randomized Studies of Interventions tool (ROBINS‐I) for non‐randomized studies, an adapted five‐star Newcastle–Ottawa Scale (NOS) for cohort studies (scores ≤3 indicate high risk of bias) and an adapted eight‐star NOS for repeat cross‐sectional studies (higher scores indicate lower risk of bias).

*Participants:* All either <18 years (except one study, aged 11–19 years [34]) and/or school students from the USA. Sample sizes ranged from 14 [33] to 10 758 [30].

*Interventions:* Except one with insufficient information [39], all interventions communicated the absolute harms of vaping (e.g. causes diseases, lung injuries) and risks of nicotine, mainly with the aim of deterring young people from vaping. Three also communicated inaccurate relative harm (vaping is equally/more harmful than smoking) [32, 33, 34] and none communicated accurate relative harms. Interventions were mainly educational workshops/programmes delivered in schools [28, 30, 31, 35, 38, 39] but also included video games [27, 32, 33, 37], mass media campaigns (either as part of an experimental paradigm or self‐reported exposure) [29, 34, 36] or written materials [26].

*Outcomes:* All 14 articles assessed absolute harm perceptions [26, 27, 28, 29, 30, 31, 32, 33, 34, 35, 36, 37, 38, 39], two articles additionally examined relative harm perceptions [26, 30] and four articles examined nicotine harm perceptions [26, 27, 28, 29]. Most (10 articles) assessed outcome(s) pre‐ and immediately post‐intervention, with no long‐term follow‐up [26, 28, 30, 31, 32, 33, 34, 36, 37, 38], although one cohort study assessed outcome(s) at baseline and again at 13 months [29].

*Interventions and absolute harm perceptions:* Fourteen articles were identified, of which 12 found some evidence of associations (i.e. *P* < 0.05). Specifically, absolute harm perceptions were increased by: written information [26] and educational workshops/programmes [28, 30, 31, 35], highlighting that vaping and nicotine are harmful; and virtual reality video games [27, 32, 33, 37] and self‐reported or forced exposure to mass media campaigns [29, 34, 36], highlighting that vaping and nicotine are harmful and also that vaping is equally/more harmful than smoking. The two studies that did not find significant associations with absolute harm perceptions involved educational programmes delivered in schools communicating vaping and nicotine harms, although these ‘harms’ were not defined [38, 39].

*Interventions and relative harm perceptions:* Two studies were identified, both of which found some evidence that inaccurate relative perceptions were increased by written information [26] and an educational programme [30] communicating the harmful effects of vaping and nicotine.

*Interventions and nicotine harm perceptions:* Four studies were identified, three of which found some evidence for associations with interventions. Specifically, an educational programme [28] and virtual reality video game [27] communicating information that vaping is harmful and nicotine is addictive increased nicotine harm and addictiveness perceptions, one study found some evidence that exposure to ‘The Real Cost’ campaign in the USA (with graphic videos of the health consequences of vaping and addiction) increased nicotine harm perceptions on three of eight measures 13 months later [29], and one study lacked clear evidence of an effect (i.e. *P* > 0.05) that written information about vaping harms and nicotine changed the perception that e‐cigarettes contain addictive nicotine [26].

#### Adults

Among adults, 32 articles assessed associations between interventions and changes in vaping harm perceptions (Table 3) [40, 41, 42, 43, 44, 45, 46, 47, 48, 49, 50, 51, 52, 53, 54, 55, 56, 57, 58, 59, 60, 61, 62, 63, 64, 65, 66, 67, 68, 69, 70, 71]. Three articles used data from the same study [63, 64, 66].

**TABLE 3:** Interventions communicating the harms of vaping and associations with changes in vaping harm perceptions (research question 1) among adults (*n* = 32 articles). Findings are sorted by number of outcomes assessed followed by finding an effect of the intervention on an outcome.

Ref., country	Analytic sample, design	Interventions (message)		Outcomes (harm perceptions): measurement and association with intervention			Risk of bias^b^
		Category^a^	Details and examples	Relative (accurate)^a^	Absolute^a^	Nicotine^a^	
Tan (2015) [40] USA	*n* = 411 Mean age 20 years Cohort, 3‐month follow‐up	Relative – accurate and inaccurate Absolute Nicotine	Self‐reported exposure to: (1) incorrect claims about vaping (e.g. cause lung cancer, just as dangerous as cigarettes, nicotine may still contribute to heart disease, contains tar, contains carcinogens); (2) information refuting incorrect claims about vaping (i.e. refuting the claims above)	**✓**/✘ Perceptions of vaping harm and nicotine risk (cause lung cancer, just as dangerous as cigarettes, nicotine may still contribute to heart disease, contains tar, contains carcinogens) decreased after self‐reported exposure to refuting incorrect information (condition 2) but not exposure to incorrect claims about vaping (condition 1)			NOS: 2/5 (high risk)
Lee (2018) [41] USA	*n* = 666 Age 18–25 years Young adults Randomized experiment	Absolute Nicotine	Written warning labels (two conditions): (1) FDA (‘WARNING: This product contains nicotine derived from tobacco. Nicotine is an addictive chemical.”); (2) e‐cigarette company (e.g. ‘This product is not a smoking cessation product … intended for use by persons of legal age or older … nicotine is addictive and habit forming, and it is very toxic by inhalation … may aggravate existing respiratory conditions)	**✓/**✘ Perceptions of absolute harm and nicotine addiction (die prematurely, damage overall health, damage lungs, and developing lung cancer, heart disease, mouth/teeth problems and nicotine addiction) increased following FDA but not e‐cigarette company labelling. Perceptions that vaping is less harmful than smoking (relative) can help smokers quit, and is less addictive than smoking, did not change after either condition			RoB2: some concerns
Ajumobi (2022) [42] USA	*n* = 165 Undergraduate students, age 18+ years (mean 24 years) One‐group experiment	Absolute Nicotine	Written information + smoking video. Text stating that e‐cigarettes contain nicotine, which is addictive and can harm adolescent brain, chemicals that cause cancer and respiratory problems, 68 people have died from ‘e‐cigarette smoking’. The video described the lived experience of an individual who was in recovery from tobacco smoking (note: video was not about vaping)	N/A	✓ Perceptions that e‐cigarettes are harmful/addictive (combination of three measures: harmful to the users’ health, others’ health, nicotine in e‐cigarettes/cigarettes is harmful) increased from before to immediately after the intervention		ROBINS‐I: serious risk
Grummon (2022) [43] USA	*n* = 810 Age 18+ years Smokers/vapers Randomized experiment	Absolute	Written information (social media). Six conditions comprising tweets with varying information about COVID‐19 and vaping harms (e.g. vaping increases your chances of COVID‐19; vaping damages your heart). Control messages involved neutral text about vaping (‘An e‐cigarette is a battery‐powered device that usually contains tobacco‐derived liquid. It heats the e‐liquid into an aerosol that the user inhales.’)	N/A	✓ Perceptions that vaping is harmful, increases COVID‐19 risk/death, weakens immunity, harms lungs/heart were all positively correlated with exposure to the vaping harm messages, relative to the control	✓ Perception that nicotine increases COVID‐19 risk positively correlated with exposure to the vaping harm messages, relative to the control	RoB2: some concerns
Majmun‐dar (2019) [44] USA	*n* = 191 Age 18–25 years Randomized experiment	Relative – accurate Absolute	Written information (three conditions): positive (benefits of vaping, including relative to smoking), negative (harms of vaping), ambivalent (benefits and harms)	**✓** Perceptions of vaping benefits relative to smoking (e.g. less toxic) decreased in negative and ambivalent, but not positive, conditions	**✓**/✘ No significant effect of message condition, perception that vaping is harmful decreased in all conditions	N/A	RoB2: some concerns
Yang (2018) [45] USA	*n* = 580 Age 18–64 years Current smokers Non‐randomized experiment	Relative – accurate	Written information (e.g. ‘ENDS may be as effective as the nicotine patch for helping people quit smoking’), either targeted towards the individual (smoking status and personality) or not	**✓/**✘ Perception that vaping is less harmful than smoking increased, more so for targeted versus non‐targeted messages, among some groups of smokers	**✓/**✘ Perception that vaping is harmful increased more so for targeted than non‐targeted messages among some groups of smokers	N/A	ROBINS‐I: serious risk
Liu 2022 [46] UK and USA	*n* = 2400 Age 18+ years, mean 50 years Smokers Randomized experiment	Relative – inaccurate Absolute	Written information (social media). Three conditions comprising four tweets per condition about e‐cigarette harms versus control (four tweets about physical activity). Conditions were: (1) e‐cigarettes are as or more harmful than smoking (e.g. ‘vaping is still pretty much just as dangerous as cigs’); (2) e‐cigarettes are completely harmless (misinformation, e.g. ‘there are ZERO proven harms’); (3) uncertainty (correct information (e.g. ‘We still do not know how safe vaping is’)	**✓**/✘ Compared with the control, condition 1 reduced the perception that e‐cigarettes contain fewer toxins than cigarettes and contain tar, condition 2 demonstrated no significance for perception that e‐cigarettes contain fewer toxins than cigarettes or tar; condition 3 reduced the perception that e‐cigarettes contain fewer toxins than cigarettes and contain tar	✓/✘ Compared with the control, condition 1 increased perception that e‐cigarettes contain toxic chemicals, do not contain only water vapour, contain formaldehyde, increase disease (lung cancer, heart disease, mouth/throat cancer, COPD, stroke); condition 2 reduced perception that e‐cigarettes do not cause popcorn lung but no change in other perceptions; condition 3 increased perception that e‐cigarettes contain toxic chemicals but no change in other perceptions. Condition 1 showed the greatest change	N/A	RoB2: some concerns
Oliver (2022) [47] USA	*n* = 56 Clinicians, age not reported One‐group experiment	Relative – insufficient information Absolute	Education programme. Virtual multi‐session Indiana Teen Vaping ECHO clinic, with clinicians presenting to other clinicians on diagnosing vaping dependence, teen vaping, brain effects of vaping/nicotine use and interventions to address vaping/nicotine use. Some materials included information that vaping causes lung damage and contains carcinogens	✘ Perceiving vaping as safer than smoking did not significantly change	✘ ‘Knowledge’ (definition not specified) about e‐cigarette or EVALI, the link between vaping and EVALI, and perception that vaping may increase smoking initiation, remained unchanged	N/A	ROBINS‐I: serious risk
Keating (2018) [48] USA	*n* = 192 Age 18–53 years Randomized experiment	Absolute Relative – inaccurate Nicotine	Written information (three conditions): (1) harm (‘You probably know that cigarettes contain harmful chemicals, but did you know that e‐cigarettes do as well?… e‐cigarettes contain nicotine, which is an addictive chemical’); (2) norm (‘only about 10% of [university] students say they have ever used e‐cigarettes’); (3) efficacy (‘[The university] has a ton of effective resources that can help students quit smoking’)	✘ No significant change within or between groups in perceptions that vaping is less harmful than smoking	✘ No significant change in perceptions that e‐cigarettes contain harmful chemicals, are addictive, and are bad for health across conditions	N/A	RoB2: some concerns
Calabro (2019) [49] USA	*n* = 95 Age 18–24 years Randomized experiment	Absolute Nicotine	Written messages: second‐hand vapour, dermal absorption of nicotine, nicotine addiction (e.g. ‘ppl who vape e‐cigs are haunted by nasty nicotine cravings that can take over their life!’)	N/A	**✓** Perceptions of vaping harms (‘e‐cigarettes are not a proven and safe way to quit’, ‘e‐cigarettes can lead to nicotine addiction’) increased	N/A	RoB2: some concerns
Little (2016) [50] USA	*n* = 1055 Mean age 20 years Military personnel One‐group experiment	Absolute	Educational workshop involving group discussions and motivational interviewing to improve perceived behavioural control, correct tobacco/nicotine norms, increase negative attitudes towards tobacco/nicotine use and knowledge regarding health consequences (note: no definition of knowledge or health consequences provided in the article)	N/A	**✓** Perception that vaping is harmful to health increased, while ‘do not know’ responses decreased	N/A	ROBINS‐I: serious risk
Booth (2019) [51] UK & USA	*n* = 765 Age 18–65 years Randomized experiment	Relative – accurate	Advertisements (15 advertisements): e‐cigarettes as a smoking cessation tool, healthier than cigarettes, aesthetically pleasing, celebrity endorsed, sporty, alternative to cigarettes in places where restricted, satisfying, cheaper, more fragrant, as cool as cigarettes	N/A	**✓** Perception that vaping is healthy increased overall and among smokers, but not among vapers	N/A	RoB2: some concerns
Carpenter (2021) [52] USA	*n* = 947 Age 18–30 years Smokers and/or vapers One‐group experiment	Absolute Relative – accurate Nicotine	Written messages about JUUL [e.g. for non‐smokers, JUUL could be harmful; for smokers, JUUL could be beneficial (will reduce the health harms from tobacco); one JUUL pod is equivalent to one pack of cigarettes; JUUL contains nicotine, propylene glycol, glycerine, benzoic acid.]	N/A	**✓** Perceptions of harm from using JUUL (to self and bystanders) increased	N/A	ROBINS‐I: serious risk
Kimber (2020) [53] UK	*n* = 2495 Age 18+ years Non‐vapers Randomized experiment	Nicotine Relative – accurate	Written packaging/warning labels (six conditions): (1) TPD1 (‘This product contains nicotine which is a highly addictive substance’); (2) TPD2 (‘This product contains nicotine which is a highly addictive substance. It is not recommended for non‐smokers’); (3) COMP (‘Use of this product is much less harmful than smoking’); (4) TPD1 + COMP; (5) TPD2 + COMP; (6) control (no label)	N/A	**✓** Perceptions of harm and addictiveness increased following TPD messages, more so than TPD absence conditions, but decreased following COMP alone	N/A	RoB2: low risk
DeHart (2019) [54] USA	*n* = 157 Mean age 37 years Daily smokers Randomized experiment	Relative – accurate	Written messages (four conditions): two interventions (a friend permanently switches to e‐cigarettes after a COPD diagnosis, and after physician recommends switching, and makes a recovery) and two controls	N/A	**✓** Perceptions that vaping causes diseases (e.g. lung cancer, emphysema, stroke) decreased following the two interventions. Control findings not reported	N/A	RoB2: some concerns
Pepper (2019) [55] USA	*n* = 2508 Age 18+ years Randomized experiment	Absolute	Written information (two conditions): uncertainty (e.g. ‘Not enough scientific evidence exists to say for sure how using electronic vaping products could affect your health’), control (e.g. ‘liquids in vaping products contain chemicals that are harmful when inhaled’)	N/A	**✓** Perception that vaping is harmful decreased following the uncertainty message, more so than the control	N/A	RoB2: some concerns
Popova & Ling (2014) [56] USA	*n* = 483 Age 18+ years Non‐established smokers/vapers Randomized experiment	Absolute Relative – accurate and inaccurate	Written + graphic packaging/warning labels (six conditions): (1) current (‘Warning: This product is not a safe alternative to cigarettes’); (2) graphic (mouth sore picture + ‘Warning: This product can cause mouth cancer’); (3) ‘lower risk’ (‘Warning: No tobacco product is safe, but this product presents substantially lower risks to health than cigarettes’); (4) ‘FDA approved’; (5) advertisement; (6) control (irrelevant advertisements, e.g. phone)	N/A	**✓/**✘ Perceptions of vaping harms including ‘causes cancer’ increased following condition 1 (current) and condition 2 (graphic) (including significant time–condition interaction), but no significant change for conditions 3–6	N/A	RoB2: some concerns
Prokhorov (2021) [57] USA	*n* = 446 Community college students, 18–25 years Randomized experiment	Absolute (but combined with hookah, cigarillos, snus)	Written information (SMS texts): six conditions about using e‐cigarettes, hookah, little cigars/cigarillos, snus – emotional, rational, complex, simple, loss‐framed, gain‐framed. Participants received texts on their phones for 30 days with messages	N/A	**✓/**✘ Combined risk perception of using e‐cigarettes, hookah, cigarillos, snus (five items: risk harm, risk diseases) increased overall and within all conditions, except complex and loss‐framed, at 6‐months follow‐up	N/A	RoB2: low risk
Taylor (2022) [58] USA, England, Canada, Australia	*n* = 19 005 Age 18+ years Vapers/smokers Repeat cross‐sectional (with some cohort respondents)	Nicotine	Implementation of e‐cigarette warning labels and leaflets in England, compared with USA, Canada and Australia, where no warning labels were mandated. Warning labels in England read: ‘this product contains nicotine which is a highly addictive substance’	**–**	**✓**/✘ Concern about vaping after noticing warnings did not significantly change when adjusting for covariates (demographics, smoking/vaping status, social norms) overall. However, among vapers, concern decreased after implementation	**–**	NOS: 4/5
Baer (2021) [59] USA	*n* = 41 Age 20–65 years Middle/high school staff One‐group experiment	Absolute	Educational workshop providing information about smoking and vaping among young people and associated health outcomes (e.g. heart disease, cancers, respiratory diseases)	N/A	✘ Perception that vaping affects certain health conditions did not significantly change	N/A	ROBINS‐I: serious risk
Bono (2019) [60] USA	*n* = 36 Age 18–55 years Smokers, non‐vapers One‐group experiment	Relative – accurate	Written messages (e.g. ‘reduced harm relative to cigarettes’, ‘reduced exposure to carcinogens relative to cigarettes’)	N/A	✘ Little change in perceptions of vaping harms and addictiveness (harm to health, concern about dependency)	N/A	ROBINS‐I: serious risk
Ratnes‐waran (2019) [61] UK	*n* = 106 Mean age 22 years One‐group experiment	Relative – accurate	Advertisements: five images of recent e‐cigarette advertisements: ‘a healthier option to smoking’, ‘beating the smoking ban laws’, ‘no need to quit smoking’, ‘experience the breakthrough: no tobacco, no smoke, just pure satisfaction for smokers’, ‘cigarettes, you have met your match’	N/A	✘ Perception that vaping harms health did not change	N/A	ROBINS‐I: serious risk
Patterson (2020) [62] USA	*n* = 501 Age 18–30 years LGBTQ+ women Randomized experiment	Absolute	Written information + images, with three themes: (1) health harms (e.g. ‘The truth is, vaping can damage your lungs—and you deserve to breathe freely’); (2) general wellness (e.g. ‘Strong body, strong mind, strong soul. Stay your strongest by living vape free’); (3) LGBTQ+ Pride; (4) control	N/A	✘ Perception that vaping is a ‘threat’ (harmful to health, can severely affect health, has concerning health effects) did not differ in any condition versus control	N/A	RoB2: some concerns
Yang & Popova (2020) [63]^ˠ^ USA	*n* = 1528 Age 18+ years Current/recent former smokers Randomized experiment	Relative – accurate Nicotine	Written information (four conditions): (1) comparative risk; (2) comparative risk + FDA nicotine addiction warning; (3) comparative risk + nicotine fact sheet; (4) control (bottled water advertisements)	**✓** Perceiving vaping as less harmful than smoking increased following all three comparative risk messages (1–3) more than in the control (4). Little difference among messages (1–3)	N/A	**✓**/✘ Accurate perception that nicotine is not the main cause of smoking‐related health problems decreased following nicotine fact sheet (condition 3), more than the other conditions (1, 2 and 4), but there was no change in addictiveness perceptions (nicotine main addictive substance in tobacco)	RoB2: some concerns
Yang (2020) [64] ^ˠ^ USA	*n* = 756 Age 18+ years Current/recent former smokers Randomized experiment	Nicotine	Written information (two conditions): (1) nicotine fact sheet (nicotine is not the main cause of harm from smoking, but is a poison at very high doses and is not safe to use in pregnancy, can harm the adolescent brain and is addictive); (2) control (water advertisements)	✘ No difference between pre‐ and post‐exposure in perceiving vaping as less harmful than smoking in the nicotine fact sheet versus control conditions	N/A	**✓/**✘ Accurate perception that nicotine is not the main cause of smoking‐related health problems increased following the nicotine fact sheet, more so than the control. No significant difference in perceived addictiveness for fact sheet vs control	RoB2: some concerns
Yang (2019) [65] USA	*n* = 1400 Age 18+ years Current/recent former smokers Randomized experiment	Relative – accurate	Written information (three conditions): (1) positive comparative risk (switching to e‐cigarettes can reduce health risks, with positive images, lighter colours); (2) negative comparative risk (dangers of smoking, switching to e‐cigarettes can reduce health risks, with threat‐based images and negative colours); (3) control (water advertisements)	**✓** Perception that vaping is less harmful than smoking increased in comparative risk conditions (1 and 2) more than in the control (3). No significant difference between comparative risk conditions (1 and 2)	N/A	N/A	RoB2: some concerns
Yang (2021) [66] ^ˠ^ USA	*n* = 761 Age 18+ years Current/recent former smokers Randomized experiment	Relative – accurate Nicotine	Written information (two conditions): (1) reduced risk message (switching completely to e‐cigarettes could reduce risks for smoking‐related diseases; if smokers cannot quit smoking, they can instead switch completely to e‐cigarettes) + FDA nicotine warning label (‘This product contains nicotine. Nicotine is an addictive chemical’); (2) control (bottled water advertisements)	**✓** Perceptions that switching from smoking to vaping would reduce health risks increased following intervention, more so than in the control	N/A	N/A	RoB2: some concerns
Sergakis (2019) [67] USA	*n* = 115 Age 19–36 years One‐group experiment	Relative – insufficient information Absolute	Educational video informing students about vaping health effects, prevalence and misperceptions (note: no definitions provided in the article)	**✓** Perceptions that vaping will decrease health problems caused by smoking decreased	N/A	N/A	ROBINS‐I: serious risk
Liu (2021) [68] UK and USA	*n* = 2167 Age 18+ years (mean 50 years in USA, 44 years in UK) Smokers Randomized experiment	Relative – inaccurate Absolute	Social media. Three conditions comprising four tweets per condition about e‐cigarette harms versus control (four tweets about physical activity). Conditions were: (1) e‐cigarettes are as or more harmful than smoking (misinformation, e.g. ‘vaping is still pretty much just as dangerous as cigs’); (2) e‐cigarettes are completely harmless (misinformation, e.g. ‘there are ZERO proven harms’); (3) uncertainty (correct information, e.g. ‘We still do not know how safe vaping is’)	**✓**/✘ Condition 1 (vs control) was associated with lower intention to purchase e‐cigarettes because of reduced perceptions that vaping is less harmful than smoking; condition 2 had a higher intention to purchase e‐cigarettes because of increased perceptions that vaping is less harmful than smoking; condition 3 was not significant	N/A	N/A	RoB2: some concerns
Wright (2021) [69] UK and USA	*n* = 2400 Age 18+ years, mean 50 years Smokers Randomized experiment	Relative – inaccurate Absolute	Social media. Three conditions comprising four tweets per condition about e‐cigarette harms versus control (four tweets about physical activity). Conditions were: (1) e‐cigarettes are as or more harmful than smoking (misinformation, e.g. ‘vaping is still pretty much just as dangerous as cigs’); (2) e‐cigarettes are completely harmless (misinformation, e.g. ‘there are ZERO proven harms’); (3) uncertainty (correct information, e.g. ‘We still do not know how safe vaping is’)	**✓/**✘ Condition 1 (vs control) associated with increase in the inaccurate perception that vaping is equally/more harmful than smoking; condition 2 (vs control) associated with an increase in the accurate harm perception that vaping is less harmful than smoking; condition 3 no significant association	N/A	N/A	RoB2: some concerns
Tattan‐Birch (2020) [70] UK	*n* = 1637 Age 16+ years Repeated cross‐sectional survey	Relative – accurate Nicotine	Regional mass media campaign on buses, billboards, press media, social media highlighting that vaping is less harmful than smoking, can help smokers quit and that nicotine is not responsible for most of the health harms from smoking. Control was a region with no campaign	✘ Insufficient evidence of a change in perception that vaping is less harmful than smoking	N/A	N/A	NOS: 5/8
Mungia (2022) [71] USA	*n* = 7 dental practitioners (age 32–56 years) *n* = 12 patients (age 17–26 years) One‐group experiment	Insufficient information	Educational. RAKE training for vaping cessation counselling – provides information on vaping prevalence, safety regulations, legal age of sale, mechanisms of action, chemicals, best practices and screening, evidence on oral and other health effects, evidence on vaping for tobacco cessation (but does not give details on what these are)	– No statistical comparisons, but, among dental practitioners after the intervention, perceptions that vaping is NOT less harmful and addictive than smoking increased, and perception that vaping could be a gateway to smoking increased	– No statistical comparisons, but, among dental practitioners after the intervention, perceptions that vaping is addictive and increases disease risk increased. Among patients after the intervention, perceptions that e‐cigarettes contain harmful chemicals, can harm teen brain development, have unknown long‐term health effects, are not risk‐free, and use liquid/salts made from tobacco all increased	– Among patients, the perception that e‐cigarettes usually contain nicotine, an addictive chemical, was 100% pre‐ and post‐intervention	ROBINS‐I: serious risk

✓ = evidence of associations (i.e. *P* < 0.05); ✓/✘ = mixed evidence of associations (e.g. for one subgroup only or in unadjusted but not adjusted analyses); ✘ = lacking clear evidence for an effect (i.e. *P* > 0.05); COPD = chronic obstructive pulmonary disease; EVALI = e‐cigarette or vaping use‐associated lung injury; FDA = US Food and Drug Administration; RAKE = ReACH assessment of knowledge for e‐cigarettes; SMS = Short Message Service; TPD = Tobacco Products Directive; COMP = comparative risk message.

^a^ Categories of interventions and outcomes. Relative: harms of vaping compared with smoking, subcategorized as accurate (vaping is less harmful than smoking) and inaccurate (equally/more harmful than smoking). Absolute: harms of vaping compared with not vaping. Nicotine: harms of nicotine use compared with no nicotine use.

^b^ Risk of bias was assessed using different tools depending on the study design. The Cochrane Risk of Bias (RoB) tool for randomized studies, the Risk of Bias in Non‐randomized Studies of Interventions (ROBINS‐I) tool for non‐randomized studies, an adapted five‐star Newcastle–Ottawa Scale (NOS) for cohort studies (scores ≤3 indicate high risk of bias) and an adapted eight‐star NOS for repeat cross‐sectional studies (higher scores indicate lower risk of bias).

*Participants:* Adults aged 18+ years or with a mean age of 20+ years, except one study among clinicians where age was not reported [47] and another among adults aged 16+ years [70]. Eleven studies were conducted among young adults specifically (defined above) [40, 41, 42, 44, 48, 49, 50, 52, 57, 61, 62]. The majority used US data only (24 articles) [40, 41, 42, 43, 44, 45, 47, 48, 49, 50, 52, 54, 55, 56, 57, 59, 60, 62, 63, 64, 65, 66, 67, 71], with the remainder from the UK, Canada and/or Australia, with some studies spanning more than one of these countries [46, 51, 53, 58, 61, 68, 69, 70]. Sample sizes ranged from seven [71] to 19 005 [58].

*Interventions:* Interventions among adults were more diverse than among young people, with a range of vaping harms communicated. Interventions communicating relative harms were the most common (20 articles), which included 12 accurate (less harmful than smoking) [44, 45, 51, 52, 53, 54, 60, 61, 63, 65, 66, 70], four inaccurate (equally/more harmful) [46, 48, 68, 69], two both accurate and inaccurate [40, 56] and two with insufficient information [47, 67]. The absolute harms of vaping were communicated in 18 articles [40, 42, 43, 44, 46, 47, 48, 49, 50, 52, 55, 56, 57, 59, 62, 67, 68, 69]. Nicotine harms (e.g. not the main cause of the health harms from smoking, can contribute to heart disease, is addictive) were communicated in 11 articles [40, 41, 42, 48, 49, 52, 53, 63, 64, 66, 70]. Information was mainly communicated via written materials [42, 43, 44, 45, 46, 48, 49, 52, 54, 55, 57, 60, 62, 63, 64, 65, 66, 68, 69], as well as educational workshops or videos [47, 50, 59, 67, 71], mass media campaigns [70], advertisements [51, 61] and packaging/warning labels [41, 53, 56]. One study examined self‐reported exposure to incorrect claims about vaping and exposure to information refuting these incorrect claims [40]. One article had insufficient information to classify harm perceptions into absolute, relative or nicotine perceptions [71].

*Outcomes:* Most (23 articles) examined changes in absolute harm perceptions [40, 41, 42, 43, 44, 45, 46, 47, 48, 49, 50, 51, 52, 53, 54, 55, 56, 57, 59, 60, 61, 62], 16 examined changes in relative perceptions [40, 41, 42, 44, 45, 46, 47, 48, 63, 64, 65, 66, 67, 68, 69, 70, 71] and seven examined nicotine harm perceptions [40, 41, 42, 43, 63, 64, 71]. Among these, three examined composite measures across more than one outcome group [40, 41, 42], and one assessed all three outcomes but did not report statistical comparisons [71]. Most (28 articles) assessed the outcome(s) pre‐ and immediately post‐intervention with no long‐term follow‐up [41, 42, 43, 44, 45, 46, 47, 48, 49, 50, 51, 52, 53, 54, 55, 56, 59, 60, 61, 62, 63, 64, 65, 66, 67, 68, 69, 71], although one cohort study assessed the outcome(s) at baseline and at the 3‐month follow‐up [40].

*Interventions and absolute harm perceptions:* Twenty‐three articles were identified, of which 16 found some evidence of associations. Specifically, absolute harm perceptions were changed by interventions involving written information about vaping [including social media posts, Short Message Service (SMS)/texts, written information with a video about smoking, warning labels] [41, 42, 43, 44, 45, 46, 49, 52, 53, 54, 55, 56, 57], an educational workshop [50], advertisements [51] and self‐reported exposure to incorrect claims about vaping [40]. Overall, exposure to information that vaping is harmful, causes diseases/cancer and/or is just as harmful as smoking (sometimes also in combination with information that nicotine is addictive/harmful) increased the perceptions that vaping is harmful, causes diseases/cancer and is addictive. Exposure to information that vaping does not cause cancer/diseases, is less harmful than smoking and/or helps people to quit smoking reduced perceptions that vaping is harmful, causes specific diseases and is addictive. Of the remaining articles, six lacked clear evidence that absolute harm perceptions could be changed through written information about vaping [48, 60, 62], an educational programme (one among clinicians, one among school staff) [47, 59] or advertisements [61], and one did not report statistical comparisons [71].

*Interventions and relative harm perceptions:* Sixteen articles were identified (three from the same study), of which 10 (two from the same study) found some evidence of associations. Specifically, relative harm perceptions were changed by interventions involving written information about vaping (e.g. social media, SMS/texts, warning labels, written information with images, nicotine fact sheet) [44, 45, 46, 63, 65, 66, 68, 69], an educational video [67] and self‐reported exposure to incorrect claims about vaping [40]. Overall, exposure to information that vaping is less harmful than smoking can reduce the risk of smoking‐related disease and can help people to quit smoking (sometimes also in combination with information about nicotine) increased the perception that vaping is less harmful than smoking. Exposure to information that vaping is harmful, as harmful as smoking, or that there is insufficient evidence regarding how safe vaping is increased the misperception that vaping is equally/more harmful than smoking. Among the remaining articles, five lacked clear evidence of associations between interventions and relative perceptions [41, 47, 48, 64, 70]; specifically, there was a lack of clear evidence that relative harm perceptions were changed through information about vaping and nicotine harms/addictiveness, via written information [48, 64], warning labels [41] or an educational workshop [47], or through information about the reduced harm of vaping compared with smoking provided in a regional mass media campaign [70]. The final article in this category did not report statistical comparisons [71].

*Interventions and nicotine harm perceptions:* Seven articles were identified (two from the same study), of which six (two from the same study) found some evidence of associations [40, 41, 42, 43, 63, 64]. Specifically, exposure to written information (social media posts, warning labels, written information with a video about smoking, nicotine fact sheet) that vaping is harmful, addictive, causes diseases/cancer, and that nicotine is harmful and addictive increased perceptions that nicotine is harmful and addictive and decreased the accurate perception that nicotine is not the main cause of smoking‐related harms [41, 42, 43, 63, 64]. Self‐reported exposure to information refuting incorrect claims about vaping and nicotine was associated with reduced risk perceptions of nicotine 3 months later [40]. One study did not report statistical comparisons [71].

### RQ2: do harm perceptions predict subsequent vaping and/or smoking behaviours?

#### Young people

Among young people, 14 articles assessed associations between vaping harm perceptions and changes in vaping and/or smoking behaviours (Table 4) [72, 73, 74, 75, 76, 77, 78, 79, 80, 81, 82, 83, 84, 85].

**TABLE 4:** Associations between vaping harm perceptions and subsequent changes in smoking and vaping behaviours (research question 2) among young people (*n* = 14 articles). All studies are cohort studies. Findings are sorted by number of outcomes assessed followed by finding an association between the exposure and outcome.

Ref., country	Analytic sample, approximate follow‐up	Exposure (harm perception)		Outcomes (changes in behaviour): measurement and association with exposure		Risk of bias,^b^ covariates
		Category^a^	Details and examples	Vaping	Smoking	
McKelvey (2021) [72] USA	*n* = 772 Mean age 16 years 5‐year follow‐up but outcomes assessed 2–3 years after exposure	Absolute	Perception of risks in the short and long term (scores 0–100%). Short term: addiction, nervousness, cough, colds, breathlessness, mouth sores, worse sports performance, upset friends, feeling high/buzzed, getting into trouble, bad breath. Long term: oral cancer, wrinkles, heart attack, lung cancer, another tobacco‐related illness, death	✓ Initiating (ever) or escalating (increase in number of times used) vaping predicted by lower perceptions of short−/long‐term risks of vaping	✘ Initiating (ever) and escalating (increase in number of times used) smoking	High (1) Adjusted for age
Chaffee (2018) [73] USA	*n* = 8005 Age 12–17 years 50% male, 50% female Baseline never smoked/vaped 12‐month follow‐up	Absolute	Reduction in the perception that people harm themselves when they vape between baseline and follow‐up – note that coding means we cannot determine the temporality of the exposure versus outcome.	✓ Initiating vaping (ever) associated with a reduction in perception that people harm themselves when they vape between baseline and follow‐up	✘ Initiating smoking (ever)	High (3) Demographics, sensation seeking, home tobacco use, alcohol use, other tobacco use, tobacco advertisement receptivity
Nicksic (2019) [74] USA	*n* = 5156 Age 12–17 years Never smoked/vaped at baseline 12‐month follow‐up	Relative	Perception that vaping is less harmful than smoking (vs equally/more harmful)	✓/✘ Initiating vaping (ever, but not in past 30 days) was predicted by perceiving vaping as less harmful than smoking	✘ Initiating smoking (ever; past 30 days) was not predicted by perceiving vaping as less harmful than smoking	High (3) Demographics, vaping/smoking susceptibility, sensation seeking, family tobacco use, second‐hand smoke exposure, exposure to tobacco advertising
Audrain‐McGovern (2021) [75] USA	*n* = 1808 9th grade (USA), age NR Follow‐up at 3 years	Relative	Perception of vaping harm/addictiveness (combination of two items): (1) vaping is less harmful for people to be around; and (2) vaping is less addictive than cigarettes (scored from 0, strongly disagree, to 3, strongly agree)	✘ Compared with dual vaping/smoking throughout the study, initiating vaping only and never vaping/smoking throughout the study were not associated with vaping harm/addictiveness perception. Outcome derived from latent class analysis of never, ever, past 6‐month and past 30‐day smoking/vaping		High (3) Demographics, friend/family vaping/smoking, perceived access to e‐cigarettes/cigarettes, sensation seeking, use of cigars, marijuana or alcohol, mental health, sensation seeking, expectations of vaping/smoking
Harlow (2022) [76] USA	*n* = 9584 Age 12–17 years Baseline never smoked or vaped Follow‐up at 4 years	Relative	Perception that vaping is less harmful than smoking (vs equally/more harmful, do not know)	✓ Initiating vaping (ever) was higher among those who perceived vaping as less harmful than smoking	N/A	High (3) No covariates reported
Chen‐Sankey (2019) [77] USA	*n* = 6983 Age 12–17 years Baseline never smoked/vaped 12 month follow‐up	Absolute	Perception that vaping causes some, a little or no harm (vs a lot of harm)	✓ Initiating vaping (ever; past 30 days) predicted by perceiving that vaping causes some, a little or no harm (vs a lot)	N/A	High (3) Demographics, sensation seeking, internalizing/externalizing problems
Moustafa (2021) [78] USA	*n* = 1539 Mean age 16.7 years 6‐month follow‐up	Relative	Perceived harm/addictiveness of vaping relative to smoking. Mean of two items: (1) e‐cigarettes might be less harmful for people to be around than cigarettes; (2) e‐cigarettes might be less addictive than cigarettes	✓ Increase in past 30‐day vaping (yes/no) predicted by perceiving vaping is less harmful/addictive than smoking	N/A	High (3) Demographics, past 30‐day vaping, cannabis vaping, past 6‐month smoking, peer vaping acceptance, sensation seeking
Zheng (2021) [79] USA	*n* = 6208 Age 12–17 years Follow‐up at 12 months	Absolute	Perception of how much people could harm themselves by vaping (no, a little, some, a lot)	✓ Past 30‐day vaping (yes, no; adjusting for baseline past 30‐day vaping) was predicted by lower vaping harm perceptions	N/A	High (3) Demographics, vaping status, friend vaping, physical health status
Bluestein (2022) [80] USA	*n* = 16 143 Age 12–17 years Baseline never vaped Follow up at 3–5 years	Absolute	Perception that: (1) people harm themselves when they vape (no, little harm, some harm, a lot of harm, do not know, never heard of e‐cigarettes); (2) how likely someone is to become addicted to e‐cigarettes (very/somewhat unlikely, neither, very/somewhat likely, do not know, never heard of e‐cigarettes)	✓ Earlier age of initiating vaping (ever) and first reporting past 30‐day vaping was predicted by perceiving vaping has some or a little/no, harm (vs a lot of harm) and that someone is very/somewhat unlikely to become addicted to vaping (vs very/somewhat likely). There was a dose–response relationship between earlier age of initiation and lower harm perceptions	N/A	High (2) Demographics, previous use of other tobacco products
Wang (2022) [81] USA	*n* = 8548, baseline *n* = 10 073, first follow‐up *n* = 11 641, second follow‐up (*n* in analytic sample NR) Age 12–17 years Baseline never vaped Follow‐up 12 months	Absolute	Perception of harm from vaping (no, little, some, a lot)	✓ Initiating vaping (ever, past 30 days) was predicted by perceiving less harm from vaping (no, little or some harm vs a lot of harm)	N/A	High (3) Demographics, mental health, e‐cigarette advertising exposure, parental vaping, friend vaping, current smoking, current use of other tobacco products
Parker (2018) [82] USA	*n* = 10 081 Age 12–17 years Never smoked/vaped at baseline 12‐month follow‐up	Relative Absolute	Perception that: (1) vaping is less harmful than smoking (vs equally/more harmful); (2) people harm themselves not at all, a little or some when they vape (vs a lot)	✓/✘ Initiating vaping (ever) was predicted by perceiving vaping as less harmful than smoking and that vaping poses no, a little or some harm (vs a lot). However, initiating vaping was similar among young people who perceived vaping to be about the same harmfulness as smoking versus more harmful	N/A	High (3) Demographics, alcohol use, ever tobacco use
Strong (2019) [83] USA	*n* = 10 081 (*n* = 9142 when assessing perceived risk; *n* = 9150 when assessing perceived addictiveness) Age 12–17 years 12‐month follow‐up	Relative Absolute	Perception of harm (mean of three items): (1) how much do people harm themselves when they vape; (2) how long does someone have to vape before it harms their health; (3) is vaping less/about the same/more harmful than smoking? How likely is someone to become addicted to vaping? Scored from very unlikely to very likely	✓/✘ Initiating vaping (ever) was predicted by lower perceptions that vaping is harmful/addictive	N/A	High (2) Demographics
Ahuja (2022) [84] USA	*n* = 217 Age 12–17 years Baseline vaped but not smoked in past 30 days Follow‐up at 12 months	Absolute Relative Nicotine	Perception that: (1) vaping is less harmful, about the same or more harmful than smoking; (2) people harm themselves a little/somewhat when they vape on some days but not every day (vs a lot of harm; (3) the nicotine in vapes is not at all harmful, slightly/somewhat harmful or very/extremely harmful	✓/✘ Quitting vaping predicted by (3) perceiving that nicotine in vapes is very/extremely harmful (vs slightly/somewhat) in all analyses. Quitting vaping predicted in unadjusted, but not adjusted, analyses by (1) perceiving vaping as about the same (vs less) harm as smoking; and (2) people harm themselves a lot (vs little/some) when they vape	N/A	High (3) Demographics, US census regions, other tobacco product use
Jesch (2021) [85] USA	*n* = 4470 Age 13–26 years 6‐month follow‐up	Absolute	26 items assessing beliefs about the short‐term and long‐term consequences of vaping and smoking (combined) (e.g. ‘If I vape every day, I will become addicted’)	N/A	✓ Not smoking in the past 30 days was predicted by perceiving greater consequences from vaping/smoking, with short‐term consequences more important than long‐term consequences	High (2) Demographics, baseline smoking

✓ = evidence of associations (i.e. *P* < 0.05); ✓/× = mixed evidence of associations (e.g. for one subgroup only, or in unadjusted but not adjusted analyses); ✘ = lacking clear evidence for an effect (i.e. *P* > 0.05); NR = not reported.

^a^ Categories of exposure. Relative: harm perceptions of vaping compared with smoking, subcategorized as accurate (vaping is less harmful than smoking) and inaccurate (equally/more harmful than smoking). Absolute: harm perceptions of vaping compared with not vaping. Nicotine: harm perceptions of nicotine use compared with no nicotine use.

^b^ Risk of bias was assessed for all studies using the Newcastle–Ottawa Scale (NOS), with scores of >3 stars indicating a low risk of bias and scores of ≤3 stars indicating a high risk of bias.

^c^ Same study.

*Participants:* All <18 years of age or school students, except in three studies with mean ages of 16 [72], 17 [7] or 13–26 years [85]. All studies used US data. Sample sizes ranged from 217 [84] to 16 143 [80].

*Exposures:* Vaping harm perceptions were assessed in a variety of ways, with most [10] measuring absolute harm perceptions (e.g. vaping causes diseases, wrinkles, addiction, tobacco‐related illness, is harmful to health) [72, 73, 77, 79, 80, 81, 82, 83, 84, 85], seven measuring relative harm perceptions compared with smoking [74, 75, 76, 78, 82, 83, 84] and one measuring nicotine use harm perceptions (e.g. nicotine is very/extremely harmful) [84].

*Outcomes:* Thirteen articles assessed vaping, with 12 exploring the initiation of ever and past 30‐day vaping [72, 73, 74, 75, 76, 77, 78, 79, 80, 81, 82, 83], one the escalation of vaping [72] and one quitting vaping [84]. Five articles assessed smoking, all of which assessed initiation/escalation of ever and/or past 30‐day smoking [72, 73, 74, 75, 85]. Studies could assess more than one type of exposure (perception) and outcome (behaviour). All studies were cohort surveys with outcomes measured at baseline and at follow‐ups conducted at 6 months [78, 85, 87, 108] and at 3–5 years [80].

*Absolute harm perceptions and vaping:* Nine studies were identified [72, 73, 77, 79, 80, 81, 82, 83, 84, 85], of which eight found that perceiving lower absolute harms from vaping predicted the subsequent initiation of ever and past 30‐day vaping, and the escalation of vaping [72, 73, 77, 79, 80, 81, 82, 83]. One of these studies specifically found a dose–response relationship between lower perceptions of harm from vaping and an earlier age of initiation [80]. The ninth study found that quitting vaping was predicted by perceiving that people harm themselves a little/somewhat (vs a lot) when they vape in unadjusted analyses, but not when adjusting for demographics and other tobacco product use [84].

*Absolute harm perceptions and smoking:* Three studies were identified [72, 73, 85], of which one found that perceiving greater health consequences from vaping/smoking (combined) predicted not smoking in the past 30 days [85], while two lacked clear evidence of associations between absolute vaping harm perceptions and the initiation or escalation of smoking [72, 73].

*Relative harm perceptions and vaping:* Seven studies were identified [74, 76, 78, 82, 83], of which four found that perceiving less harm from vaping relative to smoking predicted the subsequent initiation of ever vaping [74, 76, 82, 83] and the escalation of past 30‐day vaping [78], while two lacked clear evidence of these associations [74, 75]. The seventh study found that quitting vaping as an outcome was predicted by perceiving vaping to be about the same (vs less) harm as smoking in unadjusted analyses, but not when adjusting for demographics and other tobacco product use [84].

*Relative harm perceptions and smoking:* Two studies were identified, both of which lacked clear evidence that perceiving vaping as less harmful than smoking predicted the initiation of ever or past 30‐day smoking [74, 75].

*Nicotine harm perceptions and vaping:* One study was identified, which found that the perception that nicotine in vapes is very/extremely harmful was associated with subsequently quitting vaping among young people [84].

*Nicotine harm perceptions and smoking:* No studies were identified.

#### Adults

Among adults, 25 articles assessed associations between vaping harm perceptions and changes in vaping and/or smoking behaviours (Table 5) [86, 87, 88, 89, 90, 91, 92, 93, 94, 95, 96, 97, 98, 99, 100, 101, 102, 103, 104, 105, 106, 107, 108, 109, 110].

**TABLE 5:** Associations between vaping harm perceptions and subsequent changes in smoking and vaping behaviours (research question 2) among adults (*n* = 25 articles). All studies are cohort studies. Findings are sorted by number of outcomes assessed followed by finding an association between the exposure and outcome.

Ref., country	Analytic sample, approximate follow‐up	Exposure (harm perception)		Outcomes (changes in behaviour): measurement and association with exposure		Risk of bias,^b^ covariates
		Category^a^	Details and examples	Vaping	Smoking	
Kim (2022) [86] USA	*n* = 4521 (initiation), *n* = 8941 (switching), *n* = 1063 (reverting to smoking) Age 18+ years Current established smokers Follow‐up approx. 2 years	Relative	Perception that vaping is less harmful than smoking (vs equally/more harmful)	✓ Initiating vaping and completely switching to vaping were predicted by perceiving vaping as less harmful than smoking. Relapse to smoking among those who had switched less than a year ago (but not switching a year or more ago) was predicted by perceiving vaping as equally/more harmful than smoking		High (3) Demographics, time
Goldenson (2021) [87] USA, UK, Canada	*n* = 5948 (unmatched), *n* = 1132 (matched) Age 21+ years(mean 38 years USA/Canada; 33.5 years UK) Current, established smokers who had purchased JUUL Follow‐up at 6 months	Relative	Perception that using JUUL is less harmful, about the same level of harm, more harmful or much more harmful than smoking	✓/✘ Switching from smoking to JUUL was predicted by perceiving JUUL as less harmful or about the same level of harm as smoking (vs more harmful) in the unmatched, but not propensity score matched, samples		High (1) Demographics, smoking status/dependence, reason for JUUL use
Persoskie (2019) [88] USA	*n* = 2211 Age 18+ years Past 30‐day smokers and vapers Follow‐up at 12 months	Relative	Perception that vaping is less harmful than smoking (vs equally, more harmful or do not know)	✓/✘ Switching completely from smoking to vaping, past 30‐day vaping, reductions in frequency of smoking, but not frequency of vaping (changes in number of days vaped, number of puffs) was predicted by perceiving vaping as less harmful than smoking		High (2) Adjusted for demographics
Snell (2022) [89] USA	*n* = 9140 Age 18+ years Past 30‐day smokers Follow‐up approx. 4 years	Nicotine	Nicotine harm and addictiveness perceptions. Harm – composite of four measures: nicotine harms health (scored from 1, not at all, to 5, extremely), causes most of the cancer from smoking (scored from 1, definitely not, to 4, definitely yes), nicotine in cigarettes harms health (scored from 1, not at all, to 5, extremely), cigarettes with less nicotine are less, equally or more harmful than regular cigarettes. Addictiveness – composite of two measures: nicotine is the main substance in tobacco that makes people want to use it (scored from 1, definitely not, to 4, definitely yes), cigarettes with less nicotine are less, equally or more addictive than regular cigarettes	✓/✘ Past 30‐day vaping and vaping for quitting smoking was negatively predicted by perceiving nicotine as harmful, but not with addiction perceptions	✓/✘ Quit attempts were more likely, but quit success less likely, among those who perceived nicotine as harmful. There were no associations with addictiveness perceptions. Cigarette consumption was not predicted by either perception	High (3) Socio‐demographic, tobacco use, mental health, health care/access
Krishnan (2022) [90] USA	*n* = 1536 current established vapers Age 18+ years Follow‐up approx. 1 year	Absolute	Perception that vaping is harmful to health (5‐point scale from not at all harmful to extremely harmful)	✘ Continuing vaping only, smoking and continuing vaping, and smoking only (i.e. switching from vaping to smoking; vs abstinence from both) were not predicted by perceiving vaping as harmful		High (3) Demographics, smoking status, other tobacco product use, cannabis use, vape daily (vs non‐daily), vaping device type, vaping rules at home, readiness to quit vaping, advised to quit tobacco, respiratory symptoms
Romm (2022) [91] USA	n = 3006 Age 18–34 years Follow‐up at 2 years	Absolute	Perception that vaping: (1) harms health; (2) is addictive (both scored on 7‐point scale from not at all to extremely)	✓ Increases in vaping were predicted by lower perceptions of harm or addictiveness	✘ Changes in smoking were not predicted by perceptions of harm or addictiveness	High (3) Demographics
Harlow (2019) [92] USA	*n* = 6592 Age 18+ years 56% male, 44% female Current smokers, not current/experimental vapers	Relative	Perception that vaping is more harmful than smoking	✓ Not initiating current/experimental vaping was predicted by perceiving vaping as more harmful than smoking	✘ Quitting smoking was not predicted by perceiving vaping as more harmful than smoking	High (3) Demographics
Harlow (2022) [93] USA	*n* = 5699 Age 18+ years Current established smokers, not currently vaping Follow‐up approx. 2 years	Relative	Perception that vaping is less harmful than smoking (vs equally/more harmful)	✓ Initiating vaping (current) was associated with perceiving vaping as less harmful than smoking	N/A	High (3) No covariates
Brikmanis (2017) [94] USA	*n* = 348 Age 18–24 years (mean 20.5 years) Smoke at least monthly Follow‐up at 9 months	Relative	Perception that vaping is much more healthy (5) to unhealthy (1) than smoking, on a scale between 1 and 5	✓ Greater vaping frequency (proportion of days vaped in past 9 days, adjusting for baseline vaping) was predicted by perceiving vaping as more healthy than smoking	N/A	High (2)
Brose (2015) [95] UK	*n* = 1588 Age 18+ years Past‐year smokers, never vaped at baseline Follow‐up 12 months	Relative	Perception that vaping is less harmful than smoking (vs equally, more harmful or do not know)	✓ Initiating vaping (ever) was predicted by perceiving vaping as less harmful than smoking	N/A	High (3) Demographics, smoking status
Chen (2018) [96] USA	*n* = 1421 Age 18–34 years Vaped in past month Follow‐up 12 months	Relative	Perception that vaping is less harmful than smoking (v. equally or more harmful)	✓ Vaping non‐tobacco/menthol flavours (e.g. fruit) versus tobacco/menthol flavours was predicted by perceiving vaping as less harmful than smoking	N/A	High (3) Demographics, mental health, past‐month cannabis use, past‐month vaping, smoking status
Cooper (2018) [97] USA	*n* = 2565 Age 18–25 years (mean 20 years) Never vaped at baseline Follow‐up 6–24 months	Absolute	Perception that vaping: (1) harms health (not at all–extremely, 1–4); (2) is addictive (not at all–very, 1–4)	✓ Initiating vaping (ever) was predicted by lower perceived addictiveness and harms, but interactions suggested this was only significant among non‐smokers (not current smokers)	N/A	High (3) Demographics, current smoking, other tobacco use, other substance use
Elton‐Marshall et al. (2020) [98] USA	*n* = 20 628 Age 18+ years Follow‐up at 12 months	Relative	Perception that vaping is less harmful than smoking (vs equally, more harmful or do not know) and also perception that vaping is more harmful (vs equally, less or do not know)	✓ Initiating (every day/some days)/continuing vaping was predicted by perceiving vaping as less harmful than smoking	N/A	High (3) Demographics, smoking status
Yong (2014) [99] UK, Australia	*n* = 1590 Age 18+ years Current/former smokers, never vaped Follow‐up 2–3 years	Relative	Perception that vaping is less harmful than smoking (vs equally/more harmful)	✓ Initiating vaping (ever) was predicted by perceiving vaping as less harmful than smoking	N/A	High (2) Demographics, country, smoking status, interest in quitting, survey mode, wave of recruitment
Yong (2022) [100] USA, England, Canada, Australia	*n* = 1315 Age 18+ years Daily smokers at baseline who reported making a quit attempt at follow‐up Follow‐up approx. 2 years	Relative Nicotine	Perception that vaping is less harmful than smoking (vs equally/more harmful); perception that nicotine replacement therapy (NRT) is less harmful than smoking (vs equally/more harmful)	✓ Use of vapes during last attempt to quit smoking was predicted by perceiving vaping as less harmful than smoking but not by relative perceptions of NRT	N/A	High (2) Demographics, country, knowledge of smoking health effects
Choi & Forster (2014) [101] USA	*n* = 1379 Mean age 24 years Never vaped at baseline Follow‐up at 12 months	Relative	Perception that vaping is less: (1) harmful than smoking; (2) addictive than smoking (agree vs disagree/unsure)	✓/✘ Initiating vaping (ever) was predicted by perceiving vaping as less harmful than smoking, but not by perceiving vaping as less addictive than smoking	N/A	High (3) Demographics, smoking status
Hendricks (2018) [102] USA	*n* = 978 Age 19–75 years (mean 45.5 years) Current smokers Follow‐up at 6 and 12 months	Relative	Perception that vaping: (1) poses health risks relative to smoking; (2) satisfies craving/addiction relative to smoking. Calculated by subtracting scores on measure for vaping from equivalent measure for smoking (0–9, ranging from completely unlikely to completely likely)	✓/✘ Increase in vaping frequency (number of days vaped in past 30 days) was predicted by perceiving that vaping satisfies the desire for nicotine as compared with smoking (but not that vaping poses a risk to health) from 6 to 12 months, but not from 0 to 6 months	N/A	High (2) Demographics, Charlson comorbidity index, study condition
Jayakumar (2020) [103] Canada	*n* = 137 Age 16–26 years (median 17–18 years) Non‐current smoker, never vaped, at baseline Follow‐up at 12 months	Absolute Nicotine	Perception that vaping regularly without nicotine or with nicotine poses no/slight risk to long‐term health (vs moderate/great risk)	✓/✘ Initiating vaping (ever) was predicted by perceiving no/slight risk from vaping regularly without nicotine, but not with nicotine	N/A	High (2) Demographics, alcohol use, cannabis use, friends vaping, friends smoking, friends using cannabis, seeing someone vape
Vallone (2020) [104] USA	*n* = 12 114 (total sample, likely smaller for subgroup of never vapers in analysis) Age 15–34 years Follow‐up at 12 months	Relative	Perception that vaping is less harmful than smoking (vs equally/more harmful)	✓/✘ Initiating vaping (past 30‐day, but not ever, JUUL use) was predicted by perceiving vaping as less harmful than smoking	N/A	High (3) Demographics, smoking, household smoking, sensation seeking, friend vaping
Malt (2020) [105] USA	*n* = 21 693 (total sample, likely smaller for subgroup of former smokers in analysis) Age 18+ years Follow‐up at 12 months	Relative	Perception that vaping is equally/more harmful than smoking (vs less harmful)	N/A	✓ Smoking relapse (former to current smoker) was predicted by perceiving vaping as equally/more harmful than smoking	High (2)
Krishnan (2022) [106] USA	*n* = 1135 (quit attempts), *n* = 610 (quit success, among those attempting to quit) Age 18+ years Current established vapers Follow‐up approx. 1 year	Absolute	Perception that vaping is harmful to health (5‐point scale from not at all harmful to extremely harmful)	✓/✘ Quit success was predicted by greater risk perceptions of vaping. Quit attempts were predicted by greater risk perceptions of vaping in unadjusted analyses only but not when adjusting for covariates	N/A	High (3) Demographics, interest in quitting vaping, self‐efficacy to quit vaping, smoking status, other tobacco use, cannabis use, alcohol use, vaping social norms, vaping device and flavours, vaping dependence
Sobieski (2022) [107] USA	*n* = 221 Mean age 45.7 years Current vapers, not smokers Follow‐up at 2.4 years	Absolute	Perception that non‐tobacco users who try vaping are likely to become addicted (5‐point scale, from very unlikely to very likely	✘ Quit attempts and success were not predicted by perceived vaping addictiveness	N/A	High (1) No covariates adjusted for
Alalwan (2022) [108] USA	*n* = 163 Undergraduates aged 18+ years Past 30‐day JUUL users Follow‐up at 6 months	Relative	Perception that danger from, and amount of nicotine in, one JUUL pod compared with one pack of cigarettes (cigarette pack more, JUUL pod more, about the same)	✘ Attempting to quit JUUL use was not predicted by relative harm perceptions of JUUL	N/A	High (2) No covariates adjusted for
North (2021) [109] USA	*n* = 3543 Age 21–34 years Follow‐up at 1 year	Absolute	Perception that JUUL/pod vapes: (1) harm health (4‐point scale from extremely harmful to not at all harmful); (2) are addictive (3‐point scale from very to not at all)	✘ Past 30‐day pod use at follow‐up was not predicted by harm or addictiveness perceptions	N/A	High (2) Socio‐demographics, baseline vaping, baseline smoking, social norms
Wagoner (2022) [110] USA	*n* = 1534 College freshmen (mean 25.2 years) Follow‐up at 2 years	Relative	Perception that vaping is less harmful than smoking (vs equally/more harmful)	✘ Vaping initiation (ever) not predicted by perceiving vaping as less harmful than smoking (statistics provided by author, not reported in manuscript)	N/A	High (2) Socio‐demographics, smoking status, exposure to cessation claims, exposure to reduced‐risk claims

✓ = evidence of associations (i.e. *P* < 0.05); ✓/✘ = mixed evidence of associations (e.g. for one subgroup only, or in unadjusted but not adjusted analyses); ✘ = lacking clear evidence for an effect (i.e. *P* > 0.05).

^a^ Categories of exposure. Relative: harm perceptions of vaping compared with smoking, subcategorized as accurate (vaping is less harmful than smoking) and inaccurate (equally/more harmful than smoking). Absolute: harm perceptions of vaping compared with not vaping. Nicotine: harm perceptions of nicotine use compared with no nicotine use. Risk of bias was assessed for all studies using the Newcastle–Ottawa Scale (NOS), with scores of >3 stars indicating a low risk of bias and scores of ≤3 stars indicating a high risk of bias.

^b^ Risk of bias was assessed for all studies using the Newcastle Ottawa Scale (NOS), with scores of >3 stars indicating low risk of bias and scores of ≤3 stars indicating high risk of bias.

*Participants:* Most studies were conducted among adults aged 18+ years or with a mean age of 20+ years and 10 were conducted among young adults specifically (defined above) [91, 94, 96, 97, 101, 103, 104, 108, 109, 110]. The majority (20 articles) used US data only [86, 88, 89, 90, 91, 92, 93, 94, 96, 97, 98, 101, 102, 104, 105, 106, 107, 108, 109, 110], with the remainder from the UK, Canada, Australia or more than one of these countries [87, 95, 99, 100, 103]. Sample sizes ranged from 137 [103] to 20 628 [98].

*Exposures:* Unlike studies among young people, which predominantly measured absolute harm perceptions, most studies among adults measured relative vaping harm perceptions compared with smoking (17 articles) [86, 87, 88, 92, 93, 94, 95, 96, 98, 99, 100, 101, 102, 104, 105, 108, 110], seven measured absolute harm perceptions [90, 91, 97, 103, 106, 107, 109] and three measured nicotine harm perceptions [89, 100, 103].

*Outcomes:* Twenty‐two articles assessed vaping as an outcome: 11 measured the initiation of ever, past 30‐day, current, experimental and/or daily use [86, 92, 93, 95, 97, 98, 99, 101, 103, 104, 110]; six measured escalation [88, 89, 91, 94, 102, 109]; one measured vaping non‐tobacco/menthol flavours versus other flavours [96]; three measured quit attempts/success [106, 107, 108]; two measured vaping for quitting smoking [89, 100]. Six assessed smoking as an outcome, with three measuring frequency [88, 89, 91], three measuring quit attempts/success [88, 89, 92] and two measuring relapse [86, 105]. Four assessed both smoking and vaping outcomes as a combined measure, with three investigating switching from smoking to vaping [86, 87, 88] and one investigating switching from vaping to smoking [90]. Studies could assess more than one type of exposure (perception) and outcome (behaviour).

*Absolute harm perceptions and vaping:* Seven studies were identified [72, 73, 77, 79, 80, 81, 82, 83, 84], of which four found some evidence of associations [91, 97, 103, 106]. Perceiving lower absolute harms from vaping predicted subsequent vaping initiation [97, 103] and escalation [91] in three studies among young adults, while perceiving vaping as harmful to health predicted successfully quitting vaping (but not attempts to quit when adjusting for numerous covariates) in one study among adults [106]. The remaining three studies found no significant associations between absolute harm perceptions of vaping and past 30‐day pod e‐cigarette use among young adults [109], perceiving vaping as addictive and successfully quitting vaping or attempting to quit [107], and perceiving vaping as harmful and continuing vaping (vs abstinence) [90].

*Absolute harm perceptions and smoking:* Two studies were identified, neither of which found evidence that perceiving vaping as harmful or addictive predicted switching from vaping to smoking or changes in smoking [90, 91].

*Relative harm perceptions and vaping:* Sixteen studies were identified [86, 87, 88, 92, 93, 94, 95, 96, 98, 99, 100, 101, 102, 104, 105, 108, 110], of which 13 found some evidence of associations [86, 87, 88, 92, 93, 94, 95, 96, 98, 99, 100, 101, 104]. Overall, perceiving vaping as less harmful than smoking predicted: the subsequent initiation of ever and regular/current vaping among adults who smoke, as well as young adults [86, 88, 92, 93, 95, 98, 99, 101, 104]; switching from smoking to vaping [86, 87, 88]; the use of vapes during the last smoking cessation attempt [100]; past 30‐day vaping among adults who smoke [88]; and vaping non‐tobacco/menthol flavours (e.g. fruit flavours) over tobacco/menthol flavours [96]. Perceiving vaping as ‘more healthy’ than smoking also predicted increases in the frequency of vaping among young adults who smoke [94]. However, one of these studies lacked clear evidence that perceiving vaping as less harmful than smoking predicted changes in the frequency of vaping [88] and another found that associations between relative perceptions and switching from smoking to vaping were no longer significant when propensity score matching was used in the analyses [87]. The remaining three studies lacked clear evidence that relative harm perceptions predicted the initiation of ever vaping [110] or attempting to quit JUUL (a brand of e‐cigarettes that uses pods) [108] among undergraduates or increases in vaping frequency among adults who smoke [102].

*Relative harm perceptions and smoking:* Six studies were identified [86, 87, 88, 105], of which three found that perceiving vaping as less harmful than smoking predicted switching from smoking to vaping (i.e. quitting smoking) [86, 87, 88] and reductions in the frequency of smoking [88], two found that perceiving vaping as equally/more harmful than smoking predicted a relapse to smoking [86, 105], one of which found this effect was only significant among those who had recently switched [86], while one lacked clear evidence that perceiving vaping as more harmful than smoking predicted quitting smoking [92].

*Nicotine harm perceptions and vaping:* Three studies were identified [89, 100, 103], one of which found evidence of an association. Specifically, lower perceptions of nicotine as harmful predicted subsequent past 30‐day vaping and vaping for quitting smoking among adults who smoked, although addictiveness perceptions were not predictive [89]. The remaining two studies lacked clear evidence that harm perceptions of vaping with nicotine predicted the initiating of ever vaping (although vaping without nicotine was predictive of ever vaping) [103] or that perceiving nicotine replacement therapies as less harmful than smoking predicted the use of vapes during the last smoking cessation attempt [100].

*Nicotine harm perceptions and smoking:* One study was identified, which found that quit attempts were more likely, but quit success less likely, among those who perceived nicotine as harmful, and that cigarette consumption was not predicted by either nicotine harm or addictiveness perceptions [89].

### Risk of bias

The risk of bias was high overall. Most randomized studies (18 of 21) had some concerns [34, 41, 43, 44, 46, 48, 49, 51, 54, 55, 56, 62, 63, 64, 65, 66, 68, 69] and all 19 non‐randomized studies had serious concerns [26, 28, 30, 31, 32, 33, 35, 37, 38, 42, 45, 47, 50, 52, 59, 60, 61, 67, 71], mainly because of missing data or no pre‐specified analysis plan, and, for non‐randomized studies, did not adjust for key confounding factors (e.g. age or gender) (Tables S2 and S3). Fourteen of 21 randomized studies compared effects between groups [36, 43, 46, 48, 55, 56, 57, 62, 63, 64, 65, 66, 68, 69], with the remainder only assessing within‐person changes in the experimental and/or control groups. Among repeated cross‐sectional studies, the bias was average‐to‐high (with scores from 3–6 out of 8; Table S4) [39, 58, 70]. Among cohort studies, all except one (39 of 40 articles) had a high risk of bias [40, 72, 73, 74, 75, 76, 77, 78, 79, 80, 81, 82, 83, 84, 85, 86, 87, 88, 89, 90, 91, 92, 93, 94, 96, 97, 98, 99, 100, 101, 102, 103, 104, 105, 106, 107, 108, 109, 110], mainly because none had a valid measure for assessing vaping harm perceptions or bio‐verified smoking/vaping (Table S5).

## DISCUSSION

This was the first systematic review to assess, among both young people and adults: (i) what interventions have been effective in changing vaping harm perceptions; and (ii) to what extent are vaping harm perceptions predictive of any changes in vaping and smoking behaviours. Eighty‐five studies were included, of which 46 assessed interventions to change vaping harm perceptions and 39 assessed associations between harm perceptions and behaviours. All studies among young people were from the USA, and all studies among adults were from the USA, Canada, UK and/or Australia (with the majority from the USA).

A range of interventions were effective in changing vaping harm perceptions. Overall, consistent with and extending prior reviews [11, 12], the findings suggest that both young people from the USA and adults from the USA, Canada, UK and/or Australia are receptive to interventions communicating the harms of vaping, at least in the short term (38 of 46 studies only assessed the outcome pre‐ and immediately post‐intervention, with no long‐term follow‐up). Specifically, interventions communicating that vaping is harmful and addictive (absolute harm) increased these perceptions, while interventions communicating that vaping is less harmful than smoking (relative harm) increased this perception. There were also broader effects; for example, information that vaping is harmful and addictive (absolute harm) increased the misperception that vaping is as harmful as smoking (relative harm). This may, in part, explain why relative vaping misperceptions are increasing alongside more negative information about vaping in the media and some public health campaigns [4, 5, 6, 7, 8, 9, 111]. Therefore, any absolute harm messages must be balanced and carefully designed so as not to misinform people about relative harms.

There was a clear discordance between what is communicated to young people (typically absolute harm) and adults (typically relative harm). Ideally, all messages would balance the information that vaping carries risks but is less harmful than smoking, but no such studies were identified among young people. This warrants future research, particularly because young peoples’ perceptions may persist into adulthood, some communication efforts (e.g. mass media campaigns) will likely reach both groups and conflicting messages can increase inaccurate perceptions [112].

Vaping harm perceptions predicted changes in vaping and smoking behaviours, with most research focusing on vaping as an outcome. Specifically, perceiving vaping as less harmful than smoking predicted increased vaping among young people from the USA and adults from the USA, Canada, UK and/or Australia, while perceiving vaping as harmful deterred increases in vaping. These findings align with a prior meta‐analysis of mainly cross‐sectional data showing that vaping harm perceptions correlate with ever vaping among young people [13]. Our review expands this work by showing that vaping harm perceptions can precede and predict changes in both young peoples’ and adults’ vaping behaviours. With respect to smoking behaviours, the few studies available suggests that misperceiving vaping as equally/more harmful than smoking can prevent adults from switching from smoking to vaping and increase relapse risk. This is a critical finding that demonstrates the importance of promoting accurate vaping perceptions among adults who currently or formerly smoke.

The finding that accurately perceiving vaping as less harmful than smoking predicts uptake/increases in vaping among individual‐level studies is inconsistent with population‐level data showing that vaping misperceptions are increasing alongside increasing vaping prevalence in North America and the UK [5, 7, 8, 9, 113, 114]. While harm perceptions are a critical component of health behaviour, they are only one of many drivers [113, 114]. People report vaping for a variety of reasons [115, 116, 117], and so interventions that target multiple drivers of vaping and smoking, and at multiple levels (individual, local and population), are required to change these behaviours [113, 114].

The risk of bias was high overall. Some sources of bias (no pre‐specified analysis plan, missing data and a lack of adjustment for key confounding factors) can be addressed through better statistical practices. However, other sources (lack of smoking/vaping bioverification or lack of valid perception measures) are more difficult to overcome. While many biomarkers exist for smoking [118], none are validated for vaping [119] and no measures of vaping or nicotine harm perceptions have been comprehensively evaluated psychometrically. The most commonly used measure of relative perceptions (vaping is less harmful than smoking) has been assessed for criterion validity [120], but not for other reliability or validity domains. Further, as was found in this systematic review, measures vary widely, so future research should validate the range of vaping and nicotine perception measures to improve study quality in this field.

This systematic review had several limitations and implications for future research. First, as mentioned above, the risk of bias was high, limiting confidence in individual studies and therefore in the overall findings. Further, only one author assessed risk of bias, reducing the reliability of these assessments, and, because we excluded grey literature, there is also a possibility of publication bias. Second, most studies, including all studies among young people, were from the USA and may not generalize elsewhere. The USA has a unique e‐cigarette regulatory environment (e.g. no legal nicotine concentration limit on vapes and flavour restrictions in certain states) and generally negative health messaging around vaping [111], and articles spanned multiple years during which the US regulatory environment changed (e.g. the age of sale increased from 18 to 21 years in 2019). Interventions in other countries also exist [121, 122, 123] but require evaluation. Third, heterogeneity was high, with studies assessing different interventions, perceptions and behaviours; therefore, the evidence for specific interventions was often limited to a handful of studies (e.g. five for warning labels) [41, 53, 56, 58, 66]. Fourth, most intervention evaluations were limited in only assessing perceptions before and immediately after the intervention and in a controlled experimental setting. Additional research is required to assess whether the changes in perceptions are sustained in the long term. Further, in the real world, exposure to information about vaping is more complex; for example, websites/social media are the most commonly cited source of vaping communication efforts among youth [111], and vaping campaigns on social media are often accompanied by counter arguments and public reactance, which may weaken the impact of the intervention [124]. This study also has strengths. It is the largest review of its kind, considering how vaping harm perceptions can be changed, and whether they predict behaviours, among both young people and adults. Only studies with at least one time point were considered, strengthening conclusions regarding the direction of associations beyond previous reviews comprising mainly cross‐sectional studies [11, 12, 13]. Sample sizes were also large for studies assessing whether vaping harm perceptions predict behaviours.

To conclude, interventions can change vaping harm perceptions, and this is important because harm perceptions predict vaping and smoking behaviours. However, the content of interventions varies substantially, with studies among young people predominantly focusing on absolute harms (which can increase misperceptions that vaping is as harmful as smoking) and studies among adults predominantly focusing on harms relative to smoking. Ultimately, interventions should communicate that vaping carries risks but is substantially less harmful than smoking, in line with current evidence [1, 2, 3].

## AUTHOR CONTRIBUTIONS

**Katherine East:** Conceptualization; data curation; formal analysis; funding acquisition; investigation; methodology; project administration; validation; writing—original draft. **Erikas Simonavičius:** Conceptualization; data curation; formal analysis; investigation; methodology; project administration; validation; writing—review and editing. **Eve V. Taylor:** Conceptualization; data curation; formal analysis; investigation; methodology; project administration; validation; writing—review and editing. **Leonie Brose:** Conceptualization; data curation; formal analysis; funding acquisition; investigation; methodology; project administration; validation; writing—review and editing. **Deborah Robson:** Conceptualization; data curation; formal analysis; funding acquisition; investigation; methodology; project administration; validation; writing—review and editing. **Ann McNeill:** Conceptualization; data curation; formal analysis; funding acquisition; investigation; methodology; project administration; resources; supervision; validation; writing—review and editing.

## DECLARATION OF INTERESTS

D.R. is a trustee of Action on Smoking and Health. A.M., L.B. and D.R. are members of SPECTRUM, a UK Prevention Research Partnership (UKPRP) Consortium. UKPRP is an initiative funded by the UK Research and Innovation Councils, the Department of Health and Social Care (England) and the UK devolved administrations, and by leading health research charities. All other authors have no conflicts of interest to declare.

## Supporting information


**Table S1.** Preferred Reporting Items for Systematic Reviews and Meta‐Analyses (PRISMA) checklist.
**Table S2.** Risk of bias – randomised studies (RoB2).
**Table S3.** Risk of bias – non‐randomised studies (ROBINS‐I).
**Table S4.** Risk of bias – Newcastle Ottawa Scale for cross‐sectional studies.
**Table S5.** Risk of bias – Newcastle Ottawa Scale for cohort studies.

## Data Availability

Data sharing not applicable to this article because this was a review of published studies and no datasets were generated or analysed during the current study.
