# Supplementary material for: Interventions to change vaping harm perceptions and associations between harm perceptions and vaping and smoking behaviours: A systematic review
Source: Addiction. 2025 Jul 25;121(1):8–43. doi: 10.1111/add.70129 (PMC12710839; doi:10.1111/add.70129)
Supplement: Supplementary file 1 — Table S1. Preferred Reporting Items for Systematic Reviews and Meta‐Analyses (PRISMA) checklist. Table S2. Risk of bias – randomised studies (RoB2). Table S3. Risk of bias – non‐randomised studies (ROBINS‐I). Table S4. Risk of bias – Newcastle Ottawa Scale for cross‐sectional studies. Table S5. Risk of bias – Newcastle Ottawa Scale for cohort studies. [file ADD-121-8-s001.docx]

**Supporting Information**

**Table S1. Preferred Reporting Items for Systematic Reviews and Meta-Analyses (PRISMA) checklist.**

| **Section/ topic** | | **#** | | **Checklist item** | | **Location reported** | |
| --- | --- | --- | --- | --- | --- | --- | --- |
| **TITLE** | | | | | | | |
| Title | | 1 | | Identify the report as a systematic review, meta-analysis, or both. | | Title page | |
| **ABSTRACT** | | | | | | | |
| Structured summary | | 2 | | Provide a structured summary including, as applicable: background; objectives; data sources; study eligibility criteria, participants, and interventions; study appraisal and synthesis methods; results; limitations; conclusions and implications of key findings; systematic review registration number. | | Abstract | |
| **INTRODUCTION** | | | | | | | |
| Rationale | | 3 | | Describe the rationale for the review in the context of what is already known. | | Introduction | |
| Objectives | | 4 | | Provide an explicit statement of questions being addressed with reference to participants, interventions, comparisons, outcomes, and study design (PICOS). | | Introduction | |
| **METHODS** | | | | | | | |
| Protocol and registration | | 5 | | Indicate if a review protocol exists, if and where it can be accessed (e.g., Web address), and, if available, provide registration information including registration number. | | Protocol (methods) | |
| Eligibility criteria | | 6 | | Specify study characteristics (e.g., PICOS, length of follow-up) and report characteristics (e.g., years considered, language, publication status) used as criteria for eligibility, giving rationale. | | Inclusion criteria (methods) | |
| Information sources | | 7 | | Describe all information sources (e.g., databases with dates of coverage, contact with study authors to identify additional studies) in the search and date last searched. | | Search (methods) | |
| Search | | 8 | | Present full electronic search strategy for at least one database, including any limits used, such that it could be repeated. | | Supporting information: Search terms and search strategy | |
| Study selection | | 9 | | State the process for selecting studies (i.e., screening, eligibility, included in systematic review, and, if applicable, included in the meta-analysis). | | Inclusion criteria and screening (methods) | |
| Data collection process | | 10 | | Describe method of data extraction from reports (e.g., piloted forms, independently, in duplicate) and any processes for obtaining and confirming data from investigators. | | Data extraction (methods) | |
| Data items | | 11 | | List and define all variables for which data were sought (e.g., PICOS, funding sources) and any assumptions and simplifications made. | | Supporting information: Data extraction form | |
| Risk of bias in individual studies | 12 | | Describe methods used for assessing risk of bias of individual studies (including specification of whether this was done at the study or outcome level), and how this information is to be used in any data synthesis. | | Risk of bias (methods) and Tables S2-S5 (supporting information) | |  |

*Table S1 continued below.*

| **Table S1 (continued). Preferred Reporting Items for Systematic Reviews and Meta-Analyses (PRISMA) checklist** | | | |
| --- | --- | --- | --- |
| **Section/ topic** | **#** | **Checklist item** | **Location reported** |
| Summary measures | 13 | State the principal summary measures (e.g., risk ratio, difference in means). | N/A – systematic review |
| Synthesis of results | 14 | Describe the methods of handling data and combining results of studies, if done, including measures of consistency (e.g., I^2^) for each meta-analysis. | Analyses (methods) |
| Risk of bias across studies | 15 | Specify any assessment of risk of bias that may affect the cumulative evidence (e.g., publication bias, selective reporting within studies). | Risk of bias (methods) |
| Additional analyses | 16 | Describe methods of additional analyses (e.g., sensitivity or subgroup analyses, meta-regression), if done, indicating which were pre-specified. | N/A – no additional analyses |
| **RESULTS** | | | |
| Study selection | 17 | Give numbers of studies screened, assessed for eligibility, and included in the review, with reasons for exclusions at each stage, ideally with a flow diagram. | Results and Figure 1 |
| Study characterist-ics | 18 | For each study, present characteristics for which data were extracted (e.g., study size, PICOS, follow-up period) and provide the citations. | Results and Tables 2-5. |
| Risk of bias within studies | 19 | Present data on risk of bias of each study and, if available, any outcome level assessment (see item 12). | Results and Tables 2-5. |
| Results of individual studies | 20 | For all outcomes considered (benefits or harms), present, for each study: (a) simple summary data for each intervention group (b) effect estimates and confidence intervals, ideally with a forest plot. | Results and Tables 2-5. |
| Synthesis of results | 21 | Present results of each meta-analysis done, including confidence intervals and measures of consistency. | N/A – no meta-analysis |
| Risk of bias across studies | 22 | Present results of any assessment of risk of bias across studies (see Item 15). | Risk of bias (results) |
| Additional analysis | 23 | Give results of additional analyses, if done (e.g., sensitivity or subgroup analyses, meta-regression [see Item 16]). | N/A – no additional analyses |
| **DISCUSSION** | | | |
| Summary of evidence | 24 | Summarize the main findings including the strength of evidence for each main outcome; consider their relevance to key groups (e.g., healthcare providers, users, and policy makers). | Discussion |
| Limitations | 25 | Discuss limitations at study and outcome level (e.g., risk of bias), and at review-level (e.g., incomplete retrieval of identified research, reporting bias). | Discussion |
| Conclusions | 26 | Provide a general interpretation of the results in the context of other evidence, and implications for future research. | Discussion |
| **FUNDING** | | | |
| Funding | 27 | Describe sources of funding for the systematic review and other support (e.g., supply of data); role of funders for the systematic review. | Title page |

*From:* Moher D, Liberati A, Tetzlaff J, Altman DG, The PRISMA Group (2009). Preferred Reporting Items for Systematic Reviews and Meta-Analyses: The PRISMA Statement. PLoS Med 6(7): e1000097. doi:10.1371/journal.pmed1000097. [www.prisma-statement.org](http://www.prisma-statement.org).

**Search terms and search strategy**

All searches were limited to 01/01/2007 – 24/01/2023, humans.

*Medline (via PubMed):* (risk* OR harm* OR health* OR safe* OR danger* OR hazard* OR toxic* OR addict* OR damage*) AND (perception* OR perceive* OR belief* OR believe* OR attitude* OR opinion* OR approv* OR disapprov* OR accept* OR unaccept* OR aware*) AND (Electronic Cigarettes OR e-cig* OR electronic ciga* OR (ENDS AND Nicotine) OR electronic nicotine delivery system* OR (Nicotine AND (Vaping* OR Vape* OR Vapor* OR Vapouris*)))

- All fields selected, which captured keywords in all fields.

*CIHAHL (via EBSCO):* (risk* OR harm* OR health* OR safe* OR danger* OR hazard* OR toxic* OR addict* OR damage*) ) AND ( (perception* OR perceive* OR belief* OR believe* OR attitude* OR opinion* OR approv* OR disapprov* OR accept* OR unaccept* OR aware*) ) AND ( (Electronic Cigarettes OR e-cig* OR electronic ciga* OR (ENDS AND Nicotine) OR electronic nicotine delivery system* OR (Nicotine AND (Vaping* OR Vape* OR Vapor* OR Vapouris*)))

- No field selected, which captured keywords in the title, abstract, and subject headings.

*PsycInfo AND EMBASE (via Ovid):* (risk* or harm* or health* or safe* or danger* or hazard* or toxic* or addict* or damage*) and (perception* or perceive* or belief* or believe* or attitude* or opinion* or approv* or disapprov* or accept* or unaccept* or aware*) and (Electronic Cigarettes or e-cig* or electronic ciga* or (ENDS and Nicotine) or electronic nicotine delivery system* or (Nicotine and (Vaping* or Vape* or Vapor* or Vapouris*))).mp. [mp=title, abstract, heading word, drug trade name, original title, device manufacturer, drug manufacturer, device trade name, keyword, floating subheading word, candidate term word]

- Multipurpose field selected (.mp), which captured keywords in the title, abstract, subject heading, name of substance, and registry word fields.

*SCOPUS:* (risk* OR harm* OR health* OR safe* OR danger* OR hazard* OR toxic* OR addict* OR damage*) AND (perception* OR perceive* OR belief* OR believe* OR attitude* OR opinion* OR approv* OR disapprov* OR accept* OR unaccept* OR aware*) AND (Electronic Cigarettes OR e-cig* OR electronic ciga* OR (ENDS AND Nicotine) OR electronic nicotine delivery system* OR (Nicotine AND (Vaping* OR Vape* OR Vapor* OR Vapouris*)))

- All fields selected, which captured keywords in all fields.

Data extraction form (data extracted in Covidence)

**Study ID**

Lead author's name et al., publication year e.g. Goniewicz et al., 2017

**Title**

Title of paper / abstract / report that data are extracted from

**Authors**

**Year published**

**Country in which the study conducted**

1. United States
2. UK
3. Canada
4. Australia
5. Other

**Notes**

**Journal**

**Funding sources**

Extract institutions that funded research, no need to report grant numbers. NR if not reported.

**Declaration of conflicts of interest**

NR if not reported.

**RQ addressed**

1. RQ1. What interventions have been effective in changing vaping risk perceptions?
2. RQ2. To what extent are vaping risk perceptions predictive of any changes in vaping and smoking behaviours?
3. Both

**Aim of study**

A sentence summarising main aim of study

**Synopsis of main findings**

A short description of what study found

**Characteristics of included studies**

**Study design**

1. Randomised experimental study
2. Non-randomised experimental study (including one-group pre-post experiment)
3. Randomised controlled trial
4. Cohort study
5. Repeated cross sectional study
6. Crossover trial
7. Other

**Description of sample**

What participants were recruited? (e.g. general population, university students, patients, military etc.)

**Method of recruitment of participants**

1. Phone
2. Mail
3. Adverts in press
4. Adverts online
5. Clinic patients
6. Online panel
7. Other

**Sampling strategy**

1. Convenience
2. Purposive
3. Randomised
4. Quota
5. Stratified probability
6. NR
7. Other

**Research setting**

Where study has been conducted (e.g., online, research centre, hospital, university etc.)

**Data collection dates (baseline)**

mm/YYYY - mm/YYYY

**Data collection dates details**

E.g., number of survey waves or follow-ups

**Remuneration to participants**

Information on payment or other type of remuneration to participants (e.g. $40, could keep e-cigarettes after a study). NR if not reported.

**Participants**

**Inclusion criteria**

Add each criterion on a new line. NR if not reported.

**Exclusion criteria**

Add each criterion on a new line. NR if not reported.

**Total number of participants enrolled**

Number of participants who were enrolled into study

**Number of participants included in analyses**

Total number of participants in analytic sample

**Grouping of participants**

Groups and numbers of participants at baseline (e.g. intervention (n=100), control (n=100), etc.). NA if not applicable.

**Age group of sample at baseline**

1. Young people
2. Young adults
3. Adults
4. Other

**Age of sample at baseline**

Description of age of sample. E.g., age range and/or mean age. NR if not reported. If possible, extract for whole sample at baseline. If provided by group, extract by group.

**Gender of sample**

Description of gender of sample. E.g., n/% males and n/% females. NR if not reported. If possible, extract for whole sample at baseline. If provided by group, extract by group

**Race/ethnicity of sample**

Description of race/ethnicity of sample. E.g., n/% White. NR if not reported. If possible, extract for whole sample at baseline. If provided by group, extract by group.

**Other sociodemographic characteristics of sample**

Description of other sociodemographic characteristics of sample. E.g., occupation, socioeconomic status, etc. if mentioned in study. NR if not reported. If possible, extract for whole sample at baseline. If provided by group, extract by group.

**Participant smoking at baseline**

Smoking status/characteristics of sample at baseline e.g., CPD, Fagerstrom test for cigarette dependence, HSI, etc. NR if not reported. If possible, extract for whole sample at baseline. If provided by group, extract by group.

**Participant vaping at baseline**

Vaping status/characteristics of sample at baseline e.g., vaping status, frequency of vaping. NR if not reported. If possible, extract for whole sample at baseline. If provided by group, extract by group.

**RQ1 methods**

**RQ1 intervention**

**Intervention type**

1. Educational
2. Mass media
3. Advertisement
4. Packaging (including written/pictorial warning labels, imagery, alternative/experimental warnings or designs)
5. NR
6. Other

**Intervention mode**

1. Text-based (written)
2. Video
3. Photograph
4. Oral
5. NR
6. Other

**Intervention content**

What messaging of vaping did the intervention involve?

**Control group**

Was there a control group? If yes, enter brief description of the control condition. NA if there was no control group.

**Other information about intervention**

Enter other relevant information as appropriate

**RQ1 outcome**

**Type of vaping risk perception assessed**

Brief description of the type of vaping risk perceptions assessed

1. Risk of addiction
2. Risk of vaping relative to smoking
3. Risk of specific disease(s)
4. Risk of second-hand emissions
5. Risk of nicotine use
6. Risk of subsequent smoking initiation/uptake
7. Other

**Measure used**

Enter the measure wording, response options, and coding (can copy and paste from article). NR if not reported.

**Changes within- or between-person?**

Changes measured within-person (e.g., trials, experiments, longitudinal surveys) or at the population level (e.g., repeated cross-sectional surveys)?

1. Within-person (e.g., trials, pre-post experiments, longitudinal surveys)
2. Between-person (e.g., repeated cross-sectional surveys)
3. Both

**How was change assessed analytically?**

Brief description of how change in vaping risk perceptions were assessed analytically. E.g., % population increase, mean difference from Time 1 to Time 2, analyses predicting Time 2 perceptions while adjusting for Time 1/baseline perceptions

**Length of time change was assessed over**

In months. NA if pre-post experimental design.

**Other information about outcome**

Enter other relevant information as appropriate

**RQ2 methods**

**RQ2 exposure**

**Type of vaping risk perception assessed**

1. Risk of addiction
2. Risk of vaping relative to smoking
3. Risk of specific disease(s)
4. Risk of second-hand emissions
5. Risk of nicotine use
6. Risk of subsequent smoking initiation/uptake
7. Other

**Measure used**

Enter the measure wording, response options, and coding (can copy and paste from article). NR if not reported.

**Other information about exposure**

Enter other relevant information as appropriate

**RQ2 outcome**

**Outcome vaping or smoking**

1. Vaping
2. Smoking
3. Both

**Definition of vaping/smoking**

NR if not reported

**Measurement of vaping/smoking**

1. Self-report
2. Biochemically validated
3. Both
4. Other

**How was change assessed analytically?**

Brief description of how change in vaping/smoking was assessed analytically. E.g., smoking uptake among Time 1/baseline never smokers, mean difference from Time 1 to Time 2, analyses predicting Time 2 vaping while adjusting for Time 1/baseline vaping

**Length of time change was assessed over**

In months.

**Other information about outcome**

Enter other relevant information as appropriate

**Statistical analysis**

**Approach when accounting for missing data**

1. Complete case analyses
2. Imputation
3. NA - no missing data
4. NR
5. Other

**% lost to follow-up**

NR if not reported. NA if not applicable (e.g., repeated cross-sectional, pre-post experimental design)

**Statistical method**

E.g., regression, ANOVA

**Measure of effect**

1. Odds ratio
2. Risk ratio
3. Chi-squared
4. F
5. t
6. Other

**Confounders controlled for**

**Results**

**RQ1. Effect(s) of intervention and control on risk perceptions**

Sentence summarising key finding(s)

**RQ2. Association(s) between perceptions and changes in behaviour**

Sentence summarising key finding(s)

**Conclusions**

**Conclusions of study**

Sentence(s) summarising main conclusion(s) of study

**Study limitations**

Note limitations, can copy and paste from the manuscript

**Other notes**

Note anything that might have relevance when pooling findings.

**Table S2. Risk of bias – randomised studies (RoB2).**

| Study | Randomisation process | Deviations from intended interventions | Missing outcome data | Measurement of outcome | Selection of the reported result | Overall risk of bias |
| --- | --- | --- | --- | --- | --- | --- |
| Booth (2019) ^1^ | Low | Low | Low | Low | Some concerns | Some concerns |
| Calabro (2019) ^2^ | Low | Low | Low | Low | Some concerns | Some concerns |
| DeHart (2019) ^3^ | Some concerns | Low | Low | Low | Some concerns | Some concerns |
| England (2021) ^4^ | Some concerns | Low | Some concerns | Low | Some concerns | Some concerns |
| Grummon (2022) ^5^ | Some concerns | Low | Low | Low | Low | Some concerns |
| Keating (2018) ^6^ | Low | Low | Some concerns | Low | Some concerns | Some concerns |
| Kimber (2020) ^7^ | Low | Low | Low | Low | Low | Low |
| Lee (2018) ^8^ | Low | Low | Some concerns | Low | Some concerns | Some concerns |
| Liu (2021) ^9^ | Low | Low | Low | Low | Some concerns | Some concerns |
| Liu (2022) ^10^ | Low | Low | Low | Low | Some concerns | Some concerns |
| Majumdar (2019) ^11^ | Low | Low | Some concerns | Low | Some concerns | Some concerns |
| Noar (2022) ^12^ | Low | Low | Low | Low | Low | Low |
| Patterson (2020) ^13^ | Low | Low | Some concerns | Low | Some concerns | Some concerns |
| Pepper (2019) ^14^ | Low | Low | Low | Low | Some concerns | Some concerns |
| Popova (2014) ^15^ | Low | Low | Low | Low | Some concerns | Some concerns |
| Prokhorov (2021) ^16^ | Low | Low | Low | Low | Low | Low |
| Wright (2021) ^17^ | Low | Low | Low | Low | Some concerns | Some concerns |
| Yang (2020) ^18^ | Low | Low | Some concerns | Low | Some concerns | Some concerns |
| Yang (2019) ^19^ | Low | Low | Some concerns | Low | Some concerns | Some concerns |
| Yang (2020) ^20^ | Low | Low | Low | Low | Some concerns | Some concerns |
| Yang (2021) ^21^ | Low | Low | Some concerns | Low | Some concerns | Some concerns |

| Study | Confoun-ding | Selection of participants | Classifica-tion of interventions | Deviati-ons from intervention | Missing data | Measurement of outcomes | Bias in selection of reported result | Overall risk of bias |
| --- | --- | --- | --- | --- | --- | --- | --- | --- |
| Ajumobi (2022) ^22^ | Serious | Low | Low | Low | No information | Moderate | Moderate | Serious on one domain |
| Asdigian (2022) ^23^ | Serious | Serious | Low | Low | Moderate | Moderate | Moderate | Serious on one domain |
| Baer (2021) ^24^ | Serious | Low | Low | Low | No information | Moderate | Moderate | Serious on one domain |
| Baker (2022) ^25^ | Serious | Low | Low | Low | No information | Moderate | Moderate | Serious on one domain |
| Bono (2019) ^26^ | Serious | Low | Low | Low | No information | Moderate | Moderate | Serious on one domain |
| Carpenter (2021) ^27^ | Serious | Low | Low | Low | Moderate | Moderate | Moderate | Serious on one domain |
| Gaiha (2022) ^28^ | Serious | Low | Low | Low | Low | Moderate | Moderate | Serious on one domain |
| Gaiha (2021) ^29^ | Serious | Low | Low | Low | Moderate | Moderate | Moderate | Serious on one domain |
| Hieftje (2021) ^30^ | Serious | Low | Low | Low | Moderate | Moderate | Moderate | Serious on one domain |
| Little (2016) ^31^ | Serious | Low | Low | Low | No information | Moderate | Moderate | Serious on one domain |
| Merrill (2022) ^32^ | Serious | Low | Low | Low | Low | Moderate | Moderate | Serious on one domain |
| Mungia (2022) ^33^ | Serious | Low | Low | Low | Low | Moderate | Moderate | Serious on one domain |
| Noar (2019) ^34^ | Serious | Low | Low | Low | Moderate | Moderate | Moderate | Serious on one domain |
| Oliver (2022) ^35^ | Serious | Serious | Low | Low | Moderate | Moderate | Moderate | Serious on one domain |
| Pentz (2019) ^36^ | Serious | Low | Low | Low | No information | Moderate | Moderate | Serious on one domain |
| Ratneswaran (2019) ^37^ | Serious | Low | Low | Low | No information | Moderate | Moderate | Serious on one domain |
| Sergakis (2019) ^38^ | Serious | Low | Low | Low | Moderate | Moderate | Moderate | Serious on one domain |
| Weser (2021) ^39^ | Serious | Low | Low | Low | No information | Moderate | Moderate | Serious on one domain |
| Weser (2021)^40^ | Serious | Low | Low | Low | Low | Moderate | Moderate | Serious on one domain |
| Yang (2018) ^41^ | Serious | Low | Low | Low | No information | Moderate | Moderate | Serious on one domain |

**Table S3. Risk of bias – non-randomised studies (ROBINS-I).**

**Table S4. Risk of bias – Newcastle Ottawa Scale for cross-sectional studies.**

| Study | Representativeness of sample | Sample size | Non-respondents | Ascertainment of exposure | Assessment of outcome | Statistical test | Total number of stars^1^ |
| --- | --- | --- | --- | --- | --- | --- | --- |
| Levy (2022) ^42^ | 1 | 0 | 0 | 0 | 1 | 1 | 3 |
| Tattan-Birch (2020) ^43^ | 1 | 1 | 0 | 1 | 1 | 1 | 5 |
| Taylor (2022) ^44^ | 1 | 0 | 0 | 1 | 1 | 1 | 4 |

^1^ Stars are out of a maximum of 8, with higher scores indicating lower risk of bias.

**Table S5. Risk of bias – Newcastle Ottawa Scale for cohort studies.**

| Study | Representative-ness of exposed cohort | Selection of non exposed cohort | Ascertainment of exposure | Assessment of outcome | Adequacy of follow up | Risk of bias (total number of stars)^1^ |
| --- | --- | --- | --- | --- | --- | --- |
| Ahuja (2022) ^45^ | 1 | 1 | 0 | 0 | 1 | High (3) |
| Alalwan (2022) ^46^ | 0 | 1 | 0 | 0 | 1 | High (2) |
| Audrain-McGovern (2021) ^47^ | 1 | 1 | 0 | 0 | 1 | High (3) |
| Bluestein (2022) ^48^ | 1 | 1 | 0 | 0 | 0 | High (2) |
| Brikmanis (2017) ^49^ | 0 | 1 | 0 | 0 | 1 | High (2) |
| Brose (2015) ^50^ | 1 | 1 | 0 | 0 | 1 | High (3) |
| Chaffee (2018) ^51^ | 1 | 1 | 0 | 0 | 1 | High (3) |
| Chen (2018) ^52^ | 1 | 1 | 0 | 0 | 1 | High (3) |
| Chen-Sankey (2019) ^53^ | 1 | 1 | 0 | 0 | 1 | High (3) |
| Choi (2014) ^54^ | 1 | 1 | 0 | 0 | 1 | High (3) |
| Cooper (2018) ^55^ | 1 | 1 | 0 | 0 | 1 | High (3) |
| Elton-Marshall (2020) ^56^ | 1 | 1 | 0 | 0 | 1 | High (3) |
| Goldenson (2021) ^57^ | 0 | 1 | 0 | 0 | 0 | High (1) |
| Harlow (2022) ^58^ | 1 | 1 | 0 | 0 | 1 | High (3) |
| Harlow (2022) ^59^ | 1 | 1 | 0 | 0 | 1 | High (3) |
| Harlow (2019) ^60^ | 1 | 1 | 0 | 0 | 1 | High (3) |
| Hendricks (2018) ^61^ | 0 | 1 | 0 | 0 | 1 | High (2) |
| Jayakumar (2020) ^62^ | 1 | 1 | 0 | 0 | 0 | High (2) |
| Jesch (2021) ^63^ | 1 | 1 | 0 | 0 | 0 | High (2) |
| Kim (2022) ^64^ | 1 | 1 | 0 | 0 | 1 | High (3) |
| Krishnan (2022) ^65^ | 1 | 1 | 0 | 0 | 1 | High (3) |
| Krishnan (2022) ^66^ | 1 | 1 | 0 | 0 | 1 | High (3) |
| MacMonegle (2022) ^67^ | 1 | 1 | 1 | 0 | 1 | Low (4) |
| Malt (2020) ^68^ | 1 | 1 | 0 | 0 | 0 | High (2) |
| McKelvey (2021) ^69^ | 0 | 1 | 0 | 0 | 0 | High (1) |
| Moustafa (2021) ^70^ | 1 | 1 | 0 | 0 | 1 | High (3) |
| Nicksic (2019) ^71^ | 1 | 1 | 0 | 0 | 1 | High (3) |
| North (2021) ^72^ | 0 | 1 | 0 | 0 | 1 | High (2) |
| Parker (2018) ^73^ | 1 | 1 | 0 | 0 | 1 | High (3) |
| Persoskie (2019) ^74^ | 1 | 1 | 0 | 0 | 0 | High (2) |
| Romm (2022) ^75^ | 1 | 1 | 0 | 0 | 1 | High (3) |
| Snell (2022) ^76^ | 1 | 1 | 0 | 0 | 1 | High (3) |
| Sobieski (2022) ^77^ | 0 | 1 | 0 | 0 | 0 | High (1) |
| Strong (2019) ^78^ | 1 | 1 | 0 | 0 | 0 | High (2) |
| Tan (2015) ^79^ | 0 | 1 | 0 | 0 | 1 | High (2) |
| Vallone (2020) ^80^ | 1 | 1 | 0 | 0 | 1 | High (3) |
| Wagoner (2022) ^81^ | 0 | 1 | 0 | 0 | 1 | High (2) |
| Wang (2022) ^82^ | 1 | 1 | 0 | 0 | 1 | High (3) |
| Yong (2022) ^83^ | 1 | 1 | 0 | 0 | 0 | High (2) |
| Yong (2014) ^84^ | 1 | 1 | 0 | 0 | 0 | High (2) |
| Zheng (2021) ^85^ | 1 | 1 | 0 | 0 | 1 | High (3) |

^1^ Stars are out of a maximum of 5, with higher scores indicating lower risk of bias.

References

1. Booth et al. Survey of the effect of viewing an online e-cigarette advertisement on attitudes towards cigarette and e-cigarette use in adults located in the UK and USA: A cross-sectional study, BMJ Open 2019.<http://dx.doi.org/10.1136/bmjopen-2018-027525>.

2. Calabro et al. Pilot study to inform young adults about the risks of electronic cigarettes through text messaging, Addictive Behaviors Reports 2019.<http://dx.doi.org/10.1016/j.abrep.2019.100224>.

3. DeHart et al. The Experimental Tobacco Marketplace: Narratives engage cognitive biases to increase electronic cigarette substitution, Drug and Alcohol Dependence 2019.10.1016/j.drugalcdep.2019.01.020.

4. England et al. Rethink Vape: Development and evaluation of a risk communication campaign to prevent youth E-cigarette use, Addictive Behaviors 2021.<http://dx.doi.org/10.1016/j.addbeh.2020.106664>.

5. Grummon et al. Reactions to messages about smoking, vaping and COVID-19: two national experiments, ‎Tob Control 2022.10.1136/tobaccocontrol-2020-055956.

6. Keating. Extending Efforts to Move Cigarette and e-Cigarette Beliefs: Message Exposure and Belief Structures, Journal of health communication 2018.<http://dx.doi.org/10.1080/10810730.2018.1548670>.

7. Kimber et al. Communicating the relative health risks of E-cigarettes: An online experimental study exploring the effects of a comparative health message versus the EU nicotine addiction warnings on smokers' and non-smokers' risk perceptions and behavioural intentions, Addictive Behaviors 2020.10.1016/j.addbeh.2019.106177.

8. Lin et al. The effect of e-cigarette warning labels on college students' perception of e-cigarettes and intention to use e-cigarettes, Addictive Behaviors 2018.<http://dx.doi.org/10.1016/j.addbeh.2017.07.033>.

9. Liu et al. Emotional Responses and Perceived Relative Harm Mediate the Effect of Exposure to Misinformation about E-Cigarettes on Twitter and Intention to Purchase E-Cigarettes among Adult Smokers, Int J Environ Res Public Health 2021.10.3390/ijerph182312347.

10. Liu et al. Effects of brief exposure to misinformation about e-cigarette harms on Twitter on knowledge and perceptions of e-cigarettes, Digit Health 2022.10.1177/20552076221116780.

11. Majmundar et al. Examining the vulnerability of ambivalent young adults to e-cigarette messages, Health Mark Q 2020.10.1080/07359683.2019.1680119.

12. Noar et al. Impact of Vaping Prevention Advertisements on US Adolescents: A Randomized Clinical Trial, JAMA Netw Open 2022.10.1001/jamanetworkopen.2022.36370.

13. Patterson et al. Responses to e-cigarette health messages among young adult sexual minoritized women and nonbinary people assigned female at birth: Assessing the influence of message theme and format, Drug Alcohol Depend 2021.10.1016/j.drugalcdep.2021.109249.

14. Pepper et al. Impact of messages about scientific uncertainty on risk perceptions and intentions to use electronic vaping products, Addictive Behaviors 2019.<http://dx.doi.org/10.1016/j.addbeh.2018.10.025>.

15. Popova et al. Nonsmokers' responses to new warning labels on smokeless tobacco and electronic cigarettes: an experimental study, BMC Public Health 2014.<http://dx.doi.org/10.1186/1471-2458-14-997>.

16. Prokhorov et al. Mobile Text Messaging for Tobacco Risk Communication Among Young Adult Community College Students: Randomized Trial of Project Debunk, JMIR Mhealth Uhealth 2021.10.2196/25618.

17. Wright et al. Effects of brief exposure to misinformation about e-cigarette harms on twitter: a randomised controlled experiment, BMJ Open 2021.10.1136/bmjopen-2020-045445.

18. Yang et al. Communicating risk differences between electronic and combusted cigarettes: the role of the FDA-mandated addiction warning and a nicotine fact sheet, Tobacco control 2020.<http://dx.doi.org/10.1136/tobaccocontrol-2019-055204>.

19. Yang et al. Testing messages about comparative risk of electronic cigarettes and combusted cigarettes, Tobacco control 2019.<http://dx.doi.org/10.1136/tobaccocontrol-2018-054404>.

20. Yang et al. Effects of a nicotine fact sheet on perceived risk of nicotine and e-cigarettes and intentions to seek information about and use e-cigarettes, International Journal of Environmental Research and Public Health 2020.<http://dx.doi.org/10.3390/ijerph17010131>.

21. Yang et al. Will e-cigarette modified risk messages with a nicotine warning polarize smokers’ beliefs about the efficacy of switching completely to e-cigarettes in reducing smoking-related risks?, International Journal of Environmental Research and Public Health 2021.10.3390/ijerph18116094.

22. Ajumobi et al. A Brief Intervention on E-Cigarette, Regular Cigarette, and Marijuana Use Results in Generalization Effects: Lateral Attitude Change among College Students, Subst Use Misuse 2022.10.1080/10826084.2022.2086697.

23. Asdigian et al. Reducing Youth Vaping: A Pilot Test of the Peer-Led "Youth Engaged Strategies for Changing Adolescent Norms!" (YES-CAN!) Program, Health Promot Pract 2022.10.1177/15248399221100793.

24. Baer et al. Tobacco prevention education for middle school and high school educators, Health Education Journal 2021.10.1177/0017896920950344.

25. Baker et al. Vaping Prevention in a Middle School Population Using CATCH My Breath, Journal of pediatric health care : official publication of National Association of Pediatric Nurse Associates & Practitioners 2022.10.1016/j.pedhc.2021.07.013.

26. Bono et al. Effects of Electronic Cigarette Liquid Flavors and Modified Risk Messages on Perceptions and Subjective Effects of E-Cigarettes, Health Education & Behavior 2019.10.1177/1090198118806965.

27. Carpenter et al. Exploratory evaluation of online brief education for JUUL pod-mod use and prevention, Addictive Behaviors 2021.<http://dx.doi.org/10.1016/j.addbeh.2021.106942>.

28. Gaiha et al. Does virtual versus in-person e-cigarette education have a differential impact?, Health Education Journal 2022.

29. Gaiha et al. School-based e-cigarette education in Alabama: Impact on knowledge of e-cigarettes, perceptions and intent to try, Addictive Behaviors 2021.<http://dx.doi.org/10.1016/j.addbeh.2020.106519>.

30. Hieftje et al. Effectiveness of a web-based tobacco product use prevention videogame intervention on young adolescents' beliefs and knowledge, Substance Abuse 2021.10.1080/08897077.2019.1691128.

31. Little et al. Efficacy of a Brief Tobacco Intervention for Tobacco and Nicotine Containing Product Use in the US Air Force, Nicotine Tob Res 2016.10.1093/ntr/ntv242.

32. Merrill et al. A formative evaluation of an adolescent online E-cigarette prevention program, Health Education (0965-4283) 2022.10.1108/HE-06-2021-0092.

33. Mungia et al. Implementation of a youth and young adult e-cigarette cessation program within a dental clinic setting : A SToHN feasibility study, Tex Dent J 2022.

34. Noar et al. Adolescents' receptivity to E-cigarette harms messages delivered using text messaging, Addictive Behaviors 2019.10.1016/j.addbeh.2018.05.025.

35. Oliver et al. Examining the Efficacy of Project ECHO to Improve Clinicians' Knowledge and Preparedness to Treat Adolescent Vaping, Clin Pediatr (Phila) 2022.10.1177/00099228221107816.

36. Pentz et al. A videogame intervention for tobacco product use prevention in adolescents, Addictive Behaviors 2019.10.1016/j.addbeh.2018.11.016.

37. Ratneswaran et al. Electronic Cigarette Advertising Impacts Adversely on Smoking Behaviour Within a London Student Cohort: A Cross-Sectional Structured Survey, Lung 2019.<http://dx.doi.org/10.1007/s00408-019-00262-z>.

38. Sergakis et al. Evaluation of the Effects of a Brief Educational Module About Electronic Cigarettes on Undergraduate Health Professional Students’ Knowledge, Attitudes, and Self-Efficacy: A Pilot Study, Respiratory Care Education Annual 2019.

39. Weser et al. A quasi-experimental test of a virtual reality game prototype for adolescent E-Cigarette prevention, Addictive Behaviors 2021.<http://dx.doi.org/10.1016/j.addbeh.2020.106639>.

40. Weser et al. Evaluation of a virtual reality E-cigarette prevention game for adolescents, Addict Behav 2021.10.1016/j.addbeh.2021.107027.

41. Yang et al. Targeted versus nontargeted communication about electronic nicotine delivery systems in three smoker groups, International Journal of Environmental Research and Public Health 2018.<http://dx.doi.org/10.3390/ijerph15102071>.

42. Levy et al. Association of Screening and Brief Intervention With Substance Use in Massachusetts Middle and High Schools, JAMA Netw Open 2022.10.1001/jamanetworkopen.2022.26886.

43. Tattan-Birch et al. Evaluation of the Impact of a Regional Educational Advertising Campaign on Harm Perceptions of E-Cigarettes, Prevalence of E-Cigarette Use, and Quit Attempts Among Smokers, Nicotine Tob Res 2020.10.1093/ntr/ntz236.

44. Taylor et al. Changes in responses to nicotine vaping product warnings and leaflets in England compared with Canada, the US and Australia: findings from the 2016-2018 ITC Four Country Smoking and Vaping Surveys, Tobacco Control 2020.10.1136/tobaccocontrol-2020-055739.

45. Ahuja et al. Factors Associated With E-Cigarette Quitting Behavior Among Adolescents in the United States: A Prospective Observational Study, J Adolesc Health 2022.10.1016/j.jadohealth.2022.07.001.

46. Alalwan et al. Factors Associated with Quit Interest and Quit Attempts among Young Adult JUUL Users, International Journal of Environmental Research and Public Health 2022.10.3390/ijerph19031403.

47. Audrain-McGovern et al. Conjoint developmental trajectories of adolescent e-cigarette and combustible cigarette use, Pediatrics Vol 148(5), 2021, ArtID e2021051828 2021.

48. Bluestein et al. Associations Between Perceptions of e-Cigarette Harmfulness and Addictiveness and the Age of E-Cigarette Initiation Among the Population Assessment of Tobacco and Health (PATH) Youth, Tob Use Insights 2022.10.1177/1179173x221133645.

49. Brikmanis et al. E-cigarette use, perceptions, and cigarette smoking intentions in a community sample of young adult nondaily cigarette smokers, Psychol Addict Behav 2017.<http://dx.doi.org/10.1037/adb0000257>.

50. Brose et al. Perceived relative harm of electronic cigarettes over time and impact on subsequent use. A survey with 1-year and 2-year follow-ups, Drug Alcohol Depend 2015.10.1016/j.drugalcdep.2015.10.014.

51. Chaffee et al. Tobacco product initiation is correlated with cross-product changes in tobacco harm perception and susceptibility: Longitudinal analysis of the Population Assessment of Tobacco and Health youth cohort, Prev Med 2018.<http://dx.doi.org/10.1016/j.ypmed.2018.06.008>.

52. Chen et al. Prospective predictors of flavored e-cigarette use: A one-year longitudinal study of young adults in the U.S, Drug and Alcohol Dependence 2018.<http://dx.doi.org/10.1016/j.drugalcdep.2018.07.020>.

53. Chen-Sankey et al. Perceived ease of flavored e-cigarette use and e-cigarette use progression among youth never tobacco users, PLoS One 2019.<http://dx.doi.org/10.1371/journal.pone.0212353>.

54. Choi et al. Beliefs and experimentation with electronic cigarettes: a prospective analysis among young adults, Am J Prev Med 2014.10.1016/j.amepre.2013.10.007.

55. Cooper et al. A longitudinal study of risk perceptions and e-cigarette initiation among college students: Interactions with smoking status, Drug Alcohol Depend 2018.10.1016/j.drugalcdep.2017.11.027.

56. Elton-Marshall et al. Adult perceptions of the relative harm of tobacco products and subsequent tobacco product use: Longitudinal findings from waves 1 and 2 of the population assessment of tobacco and health (PATH) study, Addictive Behaviors 2020.<http://dx.doi.org/10.1016/j.addbeh.2020.106337>.

57. Goldenson et al. Differences in Switching Away From Smoking Among Adult Smokers Using JUUL Products in Regions With Different Maximum Nicotine Concentrations: North America and the United Kingdom, Nicotine Tob Res 2021.10.1093/ntr/ntab062.

58. Harlow et al. Prospective association between e-cigarette use frequency patterns and cigarette smoking abstinence among adult cigarette smokers in the United States, Addiction 2022.10.1111/add.16009.

59. Harlow et al. e-Cigarette Use and Combustible Cigarette Smoking Initiation Among Youth: Accounting for Time-Varying Exposure and Time-Dependent Confounding, Epidemiology 2022.10.1097/ede.0000000000001491.

60. Harlow et al. Socioeconomic and Racial/Ethnic Differences in E-Cigarette Uptake among Cigarette Smokers: Longitudinal Analysis of the Population Assessment of Tobacco and Health (PATH) Study, Nicotine Tob Res 2019.<http://dx.doi.org/10.1093/ntr/nty141>.

61. Hendricks et al. The Relationships of Expectancies With E-cigarette Use Among Hospitalized Smokers: A Prospective Longitudinal Study, Nicotine Tob Res 2018.10.1093/ntr/ntx043.

62. Jayakumar et al. Predictors of E-Cigarette Initiation: Findings From the Youth and Young Adult Panel Study, Tobacco Use Insights 2020.10.1177/1179173X20977486.

63. Jesch et al. Comparing belief in short-term versus long-term consequences of smoking and vaping as predictors of non-use in a 3-year nationally representative survey study of US youth, ‎Tob Control 2021.10.1136/tobaccocontrol-2021-056886.

64. Kim et al. US adult smokers' perceived relative risk on ENDS and its effects on their transitions between cigarettes and ENDS, BMC Public Health 2022.10.1186/s12889-022-14168-8.

65. Krishnan et al. Electronic Nicotine Product Cessation and Cigarette Smoking: Analysis of Waves 3 and 4 From the PATH Study, Nicotine Tob Res 2022.10.1093/ntr/ntab155.

66. Krishnan et al. Predictors of electronic nicotine product quit attempts and cessation: Analysis of waves 3 and 4 of the PATH study, Addict Behav 2022.10.1016/j.addbeh.2022.107419.

67. MacMonegle et al. Effects of a National Campaign on Youth Beliefs and Perceptions About Electronic Cigarettes and Smoking, Prev Chronic Dis 2022.10.5888/pcd19.210332.

68. Malt et al. Perception of the relative harm of electronic cigarettes compared to cigarettes amongst US adults from 2013 to 2016: Analysis of the Population Assessment of Tobacco and Health (PATH) study data, Harm Reduction Journal 2020.<http://dx.doi.org/10.1186/s12954-020-00410-2>.

69. McKelvey et al. Measures of both perceived general and specific risks and benefits differentially predict adolescent and young adult tobacco and marijuana use: findings from a Prospective Cohort Study, Humanities and Social Sciences Communications 2021.10.1057/s41599-021-00765-2.

70. Moustafa et al. Adolescent perceptions of E-cigarette use and vaping behavior before and after the EVALI outbreak, Prev Med 2021.<http://dx.doi.org/10.1016/j.ypmed.2021.106419>.

71. Nicksic et al. Is susceptibility to E-cigarettes among youth associated with tobacco and other substance use behaviors one year later? Results from the PATH study, Prev Med 2019.10.1016/j.ypmed.2019.02.006.

72. North et al. A one year prospective examination of risk factors for pod-vape use among young adults, Drug Alcohol Depend 2021.10.1016/j.drugalcdep.2021.109141.

73. Parker et al. Tobacco Product Harm Perceptions and New Use, Pediatrics 2018.10.1542/peds.2018-1505.

74. Persoskie et al. Perceived relative harm of using e‐cigarettes predicts future product switching among US adult cigarette and e‐cigarette dual users, Addiction 2019.10.1111/add.14730.

75. Romm et al. The reciprocal relationships of social norms and risk perceptions to cigarette, e-cigarette, and cannabis use: Cross-lagged panel analyses among US young adults in a longitudinal study, Drug Alcohol Depend 2022.10.1016/j.drugalcdep.2022.109570.

76. Snell et al. Associations Between Nicotine Knowledge and Smoking Cessation Behaviors Among US Adults Who Smoke, Nicotine Tob Res 2022.

77. Sobieski et al. Quitting electronic cigarettes: Factors associated with quitting and quit attempts in long-term users, Addict Behav 2022.10.1016/j.addbeh.2021.107220.

78. Strong et al. Harm perceptions and tobacco use initiation among youth in Wave 1 and 2 of the Population Assessment of Tobacco and Health (PATH) Study, Prev Med 2019.<http://dx.doi.org/10.1016/j.ypmed.2019.03.017>.

79. Tan et al. Exposure to health (mis)information: Lagged effects on young adults' health behaviors and potential pathways, Journal of Communication 2015.<http://dx.doi.org/10.1111/jcom.12163http://dx.doi.org/10.1111/jcom.12163>.

80. Vallone et al. Electronic Cigarette and JUUL Use Among Adolescents and Young Adults, JAMA Pediatrics 2020.10.1001/jamapediatrics.2019.5436.

81. Wagoner et al. Exposure to e-cigarette health claims and association with e-cigarette use and risk perceptions: A cohort study of young adults, Addictive Behaviors 2022.10.1016/j.addbeh.2022.107359.

82. Wang et al. Association of e-Cigarette Advertising, Parental Influence, and Peer Influence with US Adolescent e-Cigarette Use, JAMA Network Open 2022.10.1001/jamanetworkopen.2022.33938.

83. Yong et al. Do smokers' perceptions of the harmfulness of nicotine replacement therapy and nicotine vaping products as compared to cigarettes influence their use as an aid for smoking cessation? Findings from the ITC Four Country Smoking and Vaping Surveys, Nicotine Tob Res 2022.10.1093/ntr/ntac087.

84. Yong et al. Trends in E-Cigarette Awareness, Trial, and Use Under the Different Regulatory Environments of Australia and the United Kingdom, Nicotine Tob Res 2015.10.1093/ntr/ntu231.

85. Zheng et al. Social media and E-cigarette use among US youth: Longitudinal evidence on the role of online advertisement exposure and risk perception, Addict Behav 2021.10.1016/j.addbeh.2021.106916.
